# π-extended pyrenes: from an antiaromatic buckybowl to doubly curved nanocarbons with gulf architectures†

**DOI:** 10.1039/d4sc03460k

**Published:** 2024-09-16

**Authors:** Binbin Liu, Zhengxiong Jin, Xinyue Liu, Lanfei Sun, Cao Yang, Lei Zhang

**Affiliations:** a Beijing Advanced Innovation Center for Soft Matter Science and Engineering, Beijing University of Chemical Technology Beijing 100029 P. R. China zhl@mail.buct.edu.cn; b School of Materials Science and Engineering, The Key Laboratory of Material Processing and Mold of Ministry of Education, Henan Key Laboratory of Advanced Nylon Materials and Application, Zhengzhou University Zhengzhou 450001 P. R. China yc321@zzu.edu.cn; c Shandong North Modern Chemistry Industry Co., Ltd Jinan 252300 P. R. China

## Abstract

The synthesis of π-extended pyrenes keeps attracting considerable attention. In particular, frameworks containing nonbenzenoid rings might display intriguing properties. Here, we report a practical synthetic pathway to access a new buckybowl (1), which is composed of four five-membered rings externally fused to a pyrene core. The buckybowl 1 exhibits antiaromaticity involving 22 π-electrons, a rapid bowl-to-bowl interconversion, and a small band gap. Furthermore, this buckybowl could be subjected to Scholl cyclodehydrogenation to prepare the doubly curved nanocarbons (2_rac_ and 2_meso_), which exist as two diastereomers, as demonstrated by X-ray crystal structure determination. Variable temperature ^1^H NMR measurements reveal that 2_meso_ can isomerize into 2_rac_ under thermal conditions, with an activation free energy of 27.1 kcal mol^−1^. Both the enantiomers of 2_rac_ can be separated by chiral HPLC and their chiroptical properties are thoroughly examined. In addition, the nanocarbon 2_meso_ with two gulf architectures facilitates host–guest chemistry with a variety of guests, including PDI, TDI, C_60_ and C_70_.

## Abstract 2

An antiaromatic buckybowl and two doubly curved nanocarbons by fusion of nonbenzenoid rings onto pyrene have been synthesized and characterized.

## Graphical abstract

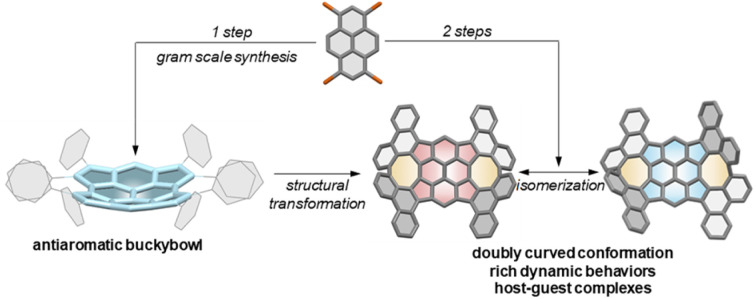

## Introduction

Incorporating nonbenzenoid rings into sp^2^ hexagonal nanographenes has emerged as a promising strategy to construct curved polycyclic aromatic hydrocarbons (PAHs).^1–5^ For example, the introduction of five-membered rings (pentagons) into a hexagonal network typically causes positive Gaussian curvature, leading to bowl-shaped PAHs (buckybowls or π-bowls),^6–12^ while seven- or eight-membered rings induce negative Gaussian curvature, leading to saddle-shaped PAHs.^13–18^ Recently, significant attention has focused on curved PAHs comprising a combination of different nonbenzenoid rings, which display both positive and negative curvatures.^19–23^ These nanostructures are attractive components of optoelectronic devices due not only to their unique electronic and photophysical properties, which result from the internal charge transfer and aromaticity between the differently sized rings,^24–27^ but also to their properties of molecular recognition towards various guests to assemble into highly ordered structures in the solid state.^28–30^ Additionally, the co-existence of both curved segments twists the molecular skeleton and induces chirality-an essential feature for some advancement in technological applications.^31^

**Fig. 1:**
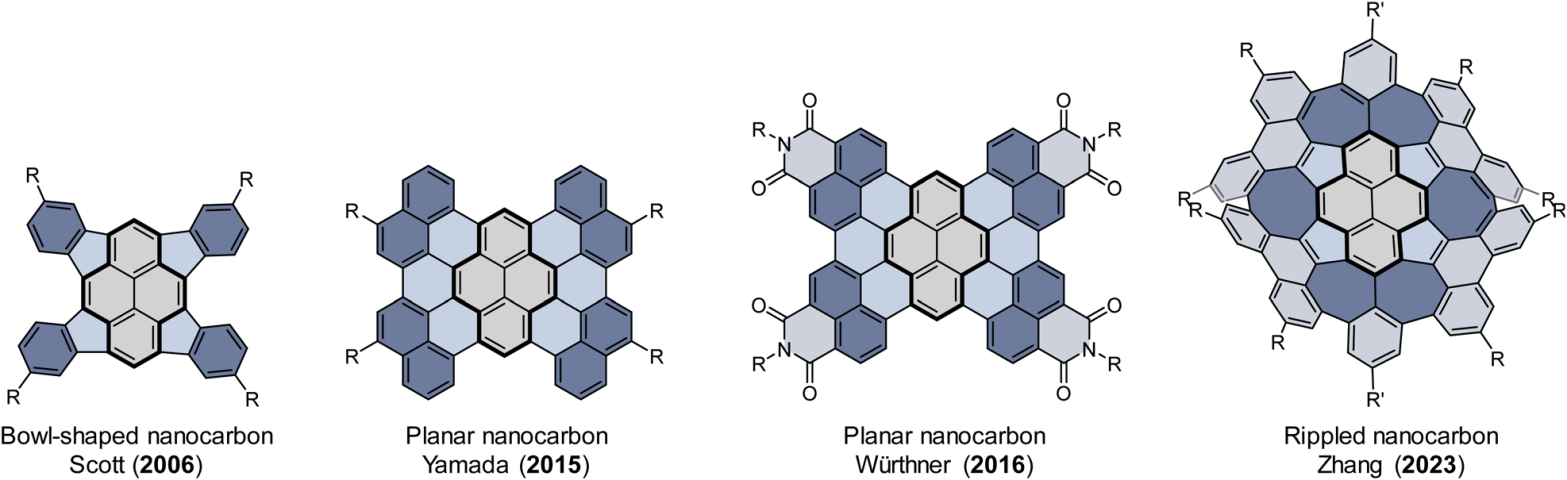
Representative of π-extended pyrenes.

**Scheme 1:**
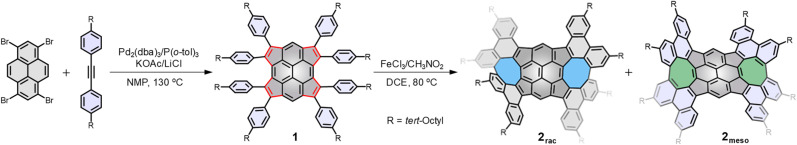
Synthesis of buckybowl 1 and doubly curved nanocarbons 2_rac_ and 2_meso_.

As the prototypical PAH, pyrene is among the most popular building blocks for complex nanocarbons.^32^ Increasing the size of pyrene by ring fusion or lateral extension of its π-system might result in novel electronic, photophysical, and supramolecular properties (Fig. 1).^33–36^ For example, Würthner reported the synthesis of a planar C64 nanographene, which is composed of four naphthalimide moieties fused to a pyrene core.^37^ This nanographene was shown to exhibit unique self-assembly with a variety of PAH guests as a result of the large π-surface and its bulky imide substituents.^38,39^ Although a growing number of π-extended pyrenes with fusion of benzenoid rings have been well-characterized,^40–46^ the π-extended pyrenes with fusion of nonbenzenoid rings are underexplored, presumably due to the σ-strain imposed on the pyrene skeleton from the externally fused nonbenzenoid rings.^47–49^ Very recently, we reported the synthesis of a rippled C84 molecular carbon, which contains ten nonbenzenoid rings that are contiguously fused to a pyrene core.^50^ These fused nonbenzenoid rings impart advantageous effects, such as high solubility, configurational stability, a narrow band gap, unique aromaticity, and ambipolar transport properties to the system. Herein we report the synthesis of a new buckybowl (1) and two doubly curved nanocarbons (2_rac_ and 2_meso_) by the fusion of nonbenzenoid rings onto the pyrene core (Scheme 1). A palladium-catalyzed cyclopentannulation of 1,3,6,8-tetrabromopyrene with diphenylacetylenes afforded tetracyclopenta[*cd*,*fg*,*jk*,*mn*]pyrene (TPP), a bowl-shaped π system that was anticipated with potentially interesting antiaromatic properties.^47,48^ We choose the diphenylacetylenes as cyclopentannulation components because the resulting pendant aryl groups at the periphery of TPP not only protect the strained double bonds from unwanted reactions but are also enabled by the application of the Scholl reaction to give the desired nanocarbons, which are structurally fascinating, but their promise for structure dynamics investigation and binding affinity towards a series of guests are perhaps of greater interest.

## Results and discussion

Scheme 1 shows the synthesis of 1, 2_rac_, and 2_meso_. The first key step was the generation of tetracyclopenta[*cd*,*fg*,*jk*,*mn*]pyrene 1, which was achieved by cyclopentannulation of 1,3,6,8-tetrabromopyrene with diphenylacetylenes by using Pd_2_(dba)_3_ as the catalyst with KOAc as the base and LiCl as the additive. This reaction process likely involves (1) reduction of the palladium(ii) salt to Pd(0), (2) oxidative addition of the aryl bromide to Pd(0), (3) arylpalladation of the alkyne to produce an open vinylic palladium intermediate, which rapidly undergoes concerted metalation deprotonation to generate the desired cyclopentadienide derivative, and (4) reductive elimination of Pd(0) for catalytic recycling (Fig. S1†). This intermediate 1 bearing sterically demanding phenyl group, allows the final step to be carried out, oxidative C–C bond formation using FeCl_3_ at 80 °C to generate two quasi-[8]circulene moieties. As expected by looking at the structure of the double quasi-[8]circulenes,^30^ two possible diastereomers, consisting of chiral (*P*,*P* and *M*,*M* configurations) and meso (*P*,*M* configuration) forms,^51^ are synthetically feasible. Fortunately, we were able to isolate and identify them. The minor and major products were identified as the *P*,*P*/*M*,*M* (2_rac_) and *M*,*P* (2_meso_) configurations, respectively, which are unambiguously verified by single-crystal X-ray analysis.

**Fig. 2:**
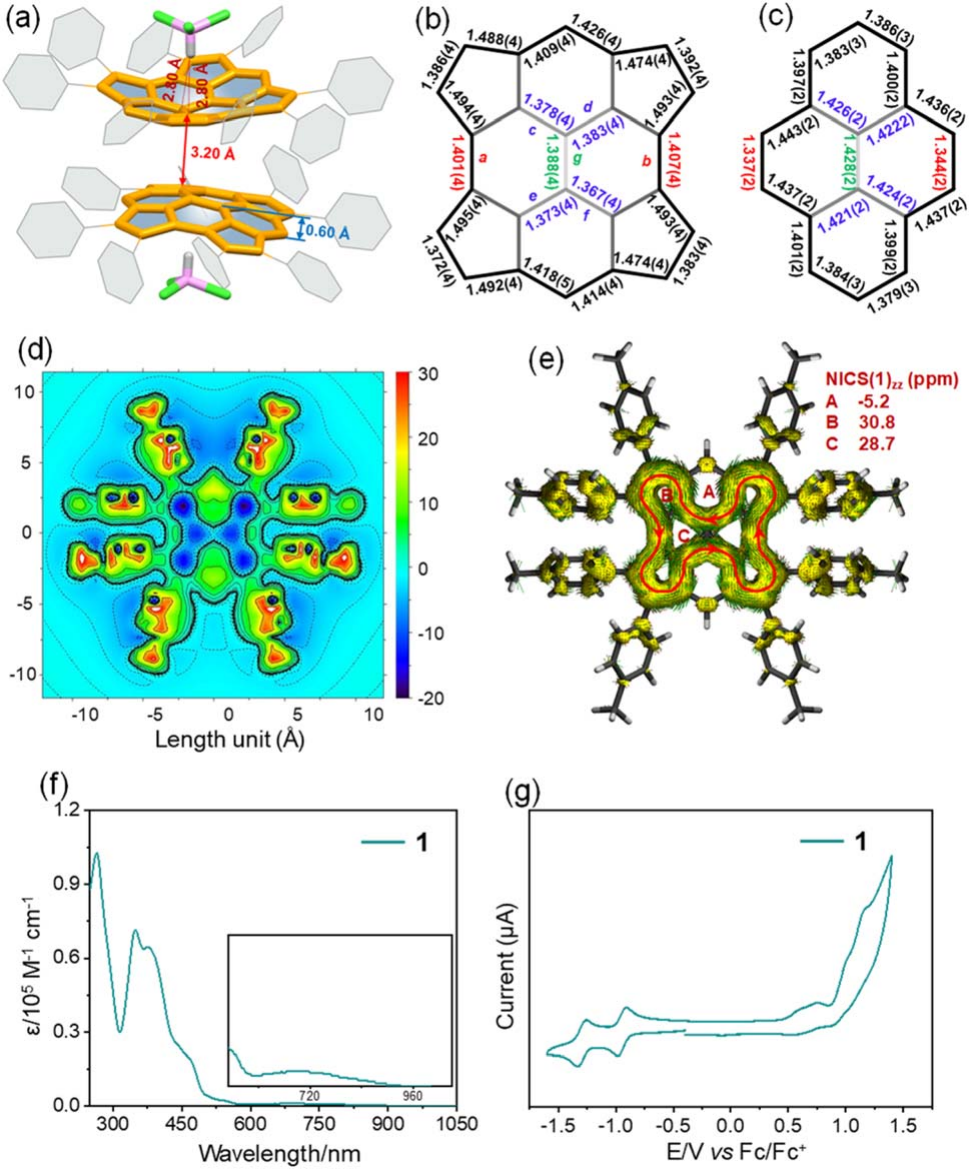
Dimeric packing motif of 1 with chloroform molecules (a) (*tert*-octyl groups and hydrogen atoms are omitted for clarity); selected bond lengths from the X-ray analysis of 1 (b) and pyrene (c); 2D ICSS_zz_ map of 1 (d); plot of calculated ACID of 1 with NICS(1)_zz_ values (e) (red circles indicate the diatropic ring currents); UV-vis absorption of 1 in chloroform (∼10^−5^ M) (f); cyclic voltammogram of 1 in nitrogen-purged dichloromethane with tetrabutylammonium hexafluorophosphate (TBAPF_6_, 0.1 M) as the supporting electrolyte and a scan rate of 100 mV s^−1^ (g).

Single crystals of 1 suitable for X-ray diffraction analysis were obtained by slow diffusion of methanol into chloroform solution. Compound 1 crystallizes with chloroform molecules and displays bowl-shaped conformation (Fig. 2a). The bowl depth is measured to be 0.60 Å based on the original 2,7-positions of the pyrene, which is shallower than that of tetraindenopyrene^36^ (0.69 Å) calculated at the B3LYP/6-311G(d,p) level of theory. The presence of chloroform molecules prevents concave–convex stacking of 1 in the crystal. Instead, the molecules adopt a concave–concave dimeric stacking model, which is based on multiple C–H⋯π (2.80 Å) and C⋯C (3.20 Å) contacts. The C–C double bonds in the five-membered rings are nearly homogeneous ranging from 1.372(4) to 1.392(4) Å, larger than that of the typical olefins (1.350 Å), and the two C–C bonds linking each of these double bonds are much longer (1.474(4)–1.495(4) Å), indicating that the C–C double bonds have a localized olefinic character and may participate only in perimeter delocalization (Fig. 2b). The bonds a and b in 1 (1.401(4) and 1.407(4) Å) are much longer than those bonds in the pyrene substructure^52^ (1.334(2) and 1.337(2) Å), while the bonds c, d, e, f, and g are observed to be considerably shorter in 1 (1.367(4)–1.388(4) Å) relative to the pyrene substructure (1.421(2)–1.428(2) Å) (Fig. 2c).

2D isochemical shielding surface (ICSS) (Fig. 2d) shows a strongly deshielded chemical environment in the inner planes of four five-membered rings and two vertical six-membered rings in 1, which are also quantified by the nucleus-independent chemical shift (NICS), suggesting the strong antiaromaticity of these rings. In line with this, the current induced density (ACID) plot (Fig. 2e) reveals a paratropic current on the periphery of 1, which involves 22 π-electrons and disobeys any of the known aromaticity rules.^53^ However, taking Clar's model into consideration, this could be explained by the fact that there are ten equal resonance structures of 1, and each contains two Clar's sextets with a local conjugated circuit involving 16 π-electrons that agrees with Hückel's rule predicting its antiaromaticity (Fig. S2 and S3†).^50^ Since they are degenerate, the resulting conjugation is their interferences and combinations. These results are also validated with the UV-vis spectrum of 1, which shows much stronger absorption in the high energy spectral region (300–400 nm), but with a broad, low energy absorbance tail that extends into 960 nm (Fig. 2f), in agreement with its antiaromatic character.^54^ Time-dependent density functional theory (TD-DFT) calculations at the B3LYP/6-31G(d,p) level of theory predict that the low-energy absorbance tail corresponds to the symmetry forbidden of the highest occupied molecular orbital (HOMO) to the lowest unoccupied molecular orbital (LUMO) transition (*λ*_calc_ = 960 nm, *f* = 0.0001) (Fig. S4 and Table S1†). Similar to cyclopenta-fused PAHs,^55^1 shows almost no emission in solution and in the solid state, likely due to the presence of the cyclopenta-fused rings in 1, which might increase the rate of intersystem crossing.^56^ In addition, 1 in dichloromethane exhibits two reversible reduction and multiple irreversible oxidation waves (Fig. 2g). The presence of two reversible reduction waves at low potentials (*E*^red^_1/2_ = −0.89 V *vs.* Fc/Fc^+^) is attributed to its high electron-accepting capacity to form cyclopentadienyl-like anions by accepting electrons, affording 4*n* + 2 aromatic systems.^57,58^

**Fig. 3:**
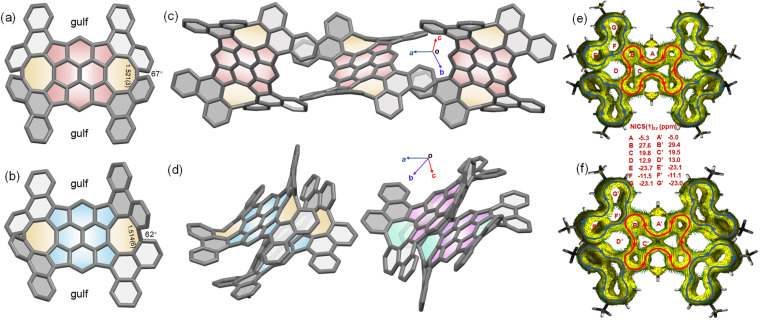
(a and b) Crystal structures of 2_rac_ and 2_meso_ with the selected bond lengths (Å) and twisted angles (*Θ*); (c and d) crystal packing of 2_rac_ and 2_meso_. Two enantiomers of 2_rac_ are in different colors (*t*-octyl groups and the solvent molecules are omitted for clarity); and (e and f) plots of calculated ACID of 2_rac_ and 2_meso_ with NICS(1)_zz_ values (blue and red circles indicate the diatropic and paratropic ring currents, respectively).

The crystals suitable for X-ray diffraction analysis were obtained by diffusion of methanol into chlorobenzene (2_meso_) and toluene (2_rac_). Both 2_rac_ and 2_meso_ display a doubly curved conformation, while much of the curvature is created by the disposition of the four phenanthrene subunits (Fig. 3a and b). In both cases, two phenanthrene subunits are directed to one side of the TPP core and the other two are directed to the other side, giving rise to the concave surfaces. The four phenanthrene subunits appear to be locked into a fixed orientation because of the steric interactions between the hydrogens of quasi-[8]circulenes. However, the central TPP cores in 2_rac_ and 2_meso_ are not bowl-shaped, as predicted by gas-phase density functional theory (DFT) calculations, but rather have a nearly planar conformation (Fig. S5†). This, however, is understandable in the TPP core because the two conformations interconvert through a vanishingly small energy barrier (Tables S2 and S3†). Thus, the preference in the crystal for the planar conformation is a result of packing forces. It is observed that the average bond lengths in TPP cores of 1, 2_rac_, and 2_meso_ are essentially identical. The torsion angles of quasi-[8]circulenes in 2_rac_ and 2_meso_ are 62° and 67°, respectively, which could account for the elongated bond lengths in quasi-[8]circulenes, leading to the weak conjugation between the neighbouring phenanthrene subunits. In the crystal of 2_meso_, the presence of gulf renders an orthogonal arrangement of the neighbouring 2_meso_, which, in turn, leads to a similar, chainlike fashion (Fig. 3c). These 2_meso_ chains pack loosely in the solid state and no any π–π interactions are observed. For 2_rac_, the crystal is a racemate composed of a 1 : 1 mixture of the enantiomers. The two neighbouring molecules adopt a homochiral dimer motif through C⋯C contacts. Each homochiral dimer engages in C–H⋯π interactions with the neighbouring enantiomeric dimers (Fig. 3d).

The calculated NICS values together with ACID plots for 2_rac_ and 2_meso_ show aromaticity in four phenanthrene subunits and antiaromaticity in the TPP core (Fig. 3e and f). Thus, five local ring currents are observed in 2_rac_ and 2_meso_, which include four diatropic currents exclusively in the four phenanthrene subunits and one paratropic current in the TPP core, consistent with the weak interaction between phenanthrene subunits. According to the NICS values, the degree of aromaticity is nearly identical in 2_rac_ and 2_meso_.

**Fig. 4:**
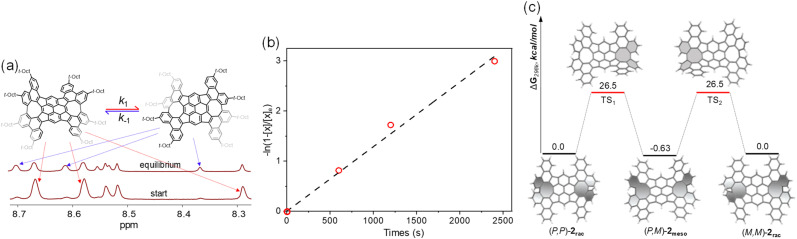
(a) Partial ^1^H NMR spectra showing isomerization of 2_meso_ to 2_rac_ at 80 °C; (b) a plot of −ln(1 – [*x*]/[*x*]_e_) for 2_meso_*versus* time; and (c) calculated diagrams for enantiomerization of (*P*,*P*)-2_meso_ and (*M*,*M*)-2_meso_.

Furthermore, a control experiment was performed to evaluate the interconversion between 2_rac_ and 2_meso_. Solution of pure 2_meso_ was prepared in 1,1,2,2-tetrachloroethane-*d*_2_ in a sealed NMR tube and heated at 80 °C, and the signals assigned to the isomer 2_rac_ began to emerge with time (Fig. 4a and S6†). After one hour, a solution containing each pure isomer comes to a final 2_meso_/2_rac_ equilibrium state, the ratio of which is determined to be 1/0.7. Accordingly, the free energy Δ*G*^‡^ for the isomerization is determined to be 1.05 kJ mol^−1^, indicating that 2_rac_ is less stable. The forward (*k*_1_) and reverse (*k*_−1_) rate constants can be estimated by using the equation −ln(1 – [*x*]/[*x*]_e_) = (*k*_1_ + *k*_−1_)t, here, [*x*] is the concentration of 2_meso_ that has been depleted at a certain time, and [*x*]_e_ is defined as [*x*] at the equilibrium state (Fig. 4b). Plotting −ln(1 – [*x*]/[*x*]_e_) *versus* time gives the equilibrium constant *k*_1_/*k*_−1_ of 1.4, and the rate constants *k*_1_ and *k*_−1_ at 80 °C of 5.3 × 10^−4^ s^−1^ and 7.6 × 10^−4^ s^−1^, respectively. Fitting the data by using the Eyring equation gives the activation free energy Δ*G*^‡^ of 26.1 kcal mol^−1^ for 2_meso_ to 2_rac_ isomerization, which is in agreement with the calculated Δ*G*^‡^ for isomerization (27.1 kcal mol^−1^). The DFT calculations also confirm that the racemization process between (*P*,*P*)-2_rac_ and (*M*,*M*)-2_rac_ proceeds *via* the intermediate (*P*,*M*)-2_meso_, with the racemization barrier of 26.5 kcal mol^−1^ (Fig. 4c and Table S4†). As expected, the racemic 2_rac_ was readily resolved into two enantiomers by chiral high-performance liquid chromatography (HPLC) at room temperature (Fig. S7†).

**Fig. 5:**
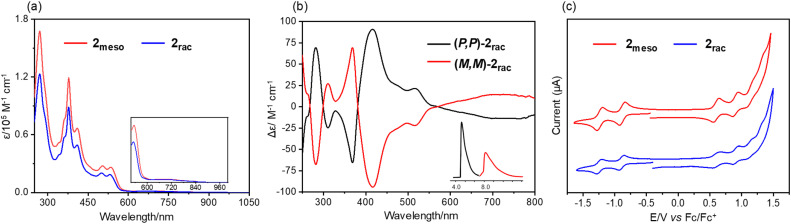
(a) UV-vis absorption of 2_rac_ and 2_meso_ in chloroform (∼10^−5^ M). (b) CD spectra of (*P*,*P*)-2_rac_ and (*M*,*M*)-2_rac_ in chloroform (inset: resolution of the enantiomers of 2_rac_ by chiral analytical HPLC). (c) Cyclic voltammograms (CV) of 2_rac_ and 2_meso_ in nitrogen-purged dichloromethane with tetrabutylammonium hexafluorophosphate (TBAPF_6_, 0.1 M) as the supporting electrolyte.

The UV-vis absorption spectrum of 2_meso_ in chloroform features four characteristic absorption peaks at 378 nm, 410 nm, 503 nm, and 535 nm (Fig. 5a). The spectrum of 2_rac_ was slightly blue-shifted but comparable to 2_meso_, with absorption peaks at 377 nm, 408 nm, 500 nm, and 530 nm. In addition, both compounds display a very low-intensity absorption tail extending to 960 nm. For 2_rac_ and 2_meso_, TD-DFT calculations suggest that the low-energy absorbance tails originate from the symmetry forbidden HOMO to LUMO transition (Tables S5 and S6†). The shorter wavelength bands for 2_rac_ and 2_meso_ are also in accordance with the simulated spectra from the TD-DFT calculations. Similar to 1, 2_rac_ and 2_meso_ show almost no emission in solution and in the solid state. Furthermore, the chiroptical properties of the enantiomers of 2_rac_ were investigated by circular dichroism (CD) measurement (Fig. 5b). The absolute configurations of the enantiomers were assigned using CD spectroscopy assisted by TD-DFT calculations (Fig. S8†). Multiple CD bands are present across the whole spectral range with |Δ*ε*| ∼ 10–85 M^−1^ cm^−1^. The Cotton effects correspond to the lowest energy absorption band with moderately strong chirooptical properties, as determined by the absorption anisotropy factor, |*g*| = |Δ*ε*|/*ε* = 0.012 at *λ* = 725 nm. Analysis of the electrochemical behaviours of 2_rac_ and 2_meso_ reveals two reversible reduction peaks, two reversible oxidation peaks, and two pseudoreversible oxidation peaks (Fig. 5c). The first oxidation and reduction potentials of 2_rac_ and 2_meso_ are almost identical, occurring at 0.50/−0.83 V and 0.53/−0.81 V (*vs.* Fc/Fc^+^), respectively, which are comparable to those of compound 1 (0.47/−0.89 V). The DFT-calculated energy levels are consistent with the trend of experimental values (Fig. S9†). Accordingly, the electrochemical HOMO–LUMO energy gaps are calculated to be 1.36 eV for 1, 1.34 eV for 2_meso_, and 1.33 eV for 2_rac_.

**Fig. 6:**
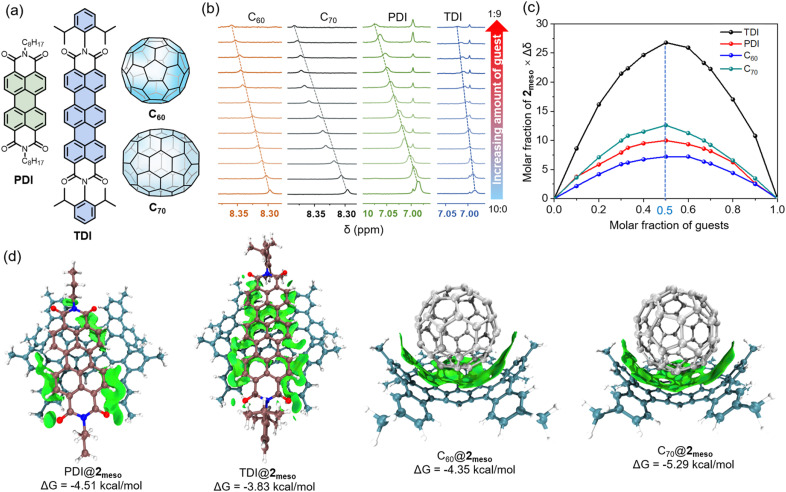
(a) Chemical structures of PDI, TDI, C_60_ and C_70_. (b) Stacked plots of partial ^1^H NMR spectra of 2_meso_ titration with different guests (the total concentration [2_meso_] + [guest] = 4.0 × 10^−4^ M, 298 K). (c) Job plots based on ^1^H NMR titration of 2_meso_ with different guests in toluene-*d*_8_ (the chemical shift change [Δ*δ*] of a proton of 2_meso_ is used and the mole fraction of guest corresponds to [guest]/[2_meso_] + [guest]). (d) Optimized superstructures and the intermolecular binding iso-surfaces of PDI@2_meso_, TDI@2_meso_, C_60_@2_meso_, and C_70_@2_meso_.

The unique gulf architecture and electron-rich concave surface of 2_meso_ indicate that this nanocarbon might be able to host a range of electron-poor guests. The planar *N*,*N*′-dioctyl-3,4,9,10-perylenedicarboximide (PDI) was selected as a representative guest (Fig. 6a). The ^1^H NMR titration revealed that both aromatic protons on 2_meso_ and PDI gradually changed upon adding PDI, indicating the presence of intermolecular interactions between 2_meso_ and PDI (Fig. 6b and S10†). The Job plot based on the titration indicates the 1 : 1 complex formation with the binding constant (*K*_a_) of 2019 M^−1^ (Fig. 6c, S11 and S12†). In addition, a 2D ^1^H–^1^H NOESY spectrum confirmed the through-space correlations between four aromatic protons of PDI and the protons of phenanthrene subunits of 2_meso_ (Fig. S13†). Furthermore, the other three guests, *N*,*N*′-di(2,6-diisopropylphenyl)-3,4,11,12 terrylenedicarboximide (TDI), C_60_, and C_70_ were selected to explore the binding ability of 2_meso_ towards guests with different shapes and electronic natures. The titration data *versus*2_meso_ for guests were fitted successfully to a 1 : 1 binding mode (Fig. 6b and c), yielding binding constants of 643 M^−1^ for TDI (Fig. S14–S16†), 1546 M^−1^ for C_60_ (Fig. S17–S19†), and 7361 M^−1^ for C_70_ (Fig. S20–S22†). Accordingly, the Gibbs free energy (Δ*G*) is calculated to be −4.51 kcal mol^−1^ for PDI@2_meso_, −3.83 kcal mol^−1^ for TDI@2_meso_, −4.35 kcal mol^−1^ for C_60_@2_meso_, and −5.29 kcal mol^−1^ for C_70_@2_meso_, respectively.

While X-ray crystal structures of the host–guest complexes could not be obtained, the relative stability of the conformers of the complexes was assessed by DFT calculations at the B3LYP/6-31G(d,p) level of theory (Fig. 6d). For PDI@2_meso_, the complex is formed by non-covalent interactions between the perylene core and the concave surface of 2_meso_, whereas in TDI@2_meso_, the terrylene core interacts with the concave surface of 2_meso_ and the concave surface of the TPP core. DFT calculations also reveal short intermolecular contacts arising from C–H⋯O interactions between the C–Hs to the alkyl chains and carbonyl groups on adjacent PDI and TDI. The octyl and 2,6-diisopropylphenyl substituents at the imide positions occupy the gulf positions to alleviate the unfavourable electrostatic interactions found in neighbouring molecules. For C_60_@2_meso_ and C_70_@2_meso_, Both C_60_ and C_70_ interact with the concave surface of 2_meso_ and the concave surface of the TPP core. In C_70_@2_meso_, 2_meso_ exhibits a preferential spatial alignment, which offers a perfect complementary shape and curvature match for the elongated side of C_70_. The distances between the centroids of the TPP core and C_60_ and C_70_ are 6.60 and 6.70 Å, respectively.

## Conclusions

In conclusion, we have reported the synthesis of a new antiaromatic buckybowl *via* a facile palladium-catalyzed cyclopentannulation. This buckybowl can be further transformed to doubly curved nanocarbons by utilizing a Scholl cyclodehydrogenation. The two quasi-[8]circulene moieties in the newly synthesized nanocarbons lead to two diastereomers (2_rac_ and 2_meso_), consisting of chiral and meso forms, respectively. DFT calculations and experimental studies reveal 2_meso_ as the more stable isomer that can isomerize into 2_rac_ at elevated temperature. The enantiomers of 2_rac_ were separated by HPLC and the chiroptical properties were investigated with CD measurement. The nanocarbons exhibit unique doubly curved conformations, which possess intriguing host–guest properties, making them attractive for both supramolecular chemistry and more complex materials. These results indicate that incorporating nonbenzenoid rings into sp^2^ carbon nanostructures is a viable strategy to not only fundamentally influence both optoelectronic and supramolecular properties but also offer extensive structure elaboration.

## Author contributions

L. Z and C. Y received and designed the project. B. L and Z. J synthesized and characterized the compounds. X. L and L. S performed DFT calculations. All authors discussed the results and contributed to the manuscript preparation.

## Conflicts of interest

There are no conflicts to declare.

## Supplementary Material

SC-015-D4SC03460K-s001

SC-015-D4SC03460K-s002

## Data Availability

Detailed synthesis and characterization of the related compounds, crystal data for 1, 2_meso_, and 2_rac_ (Table S7† and Fig. S23–S25†), ^1^H NMR, ^13^C NMR, high-resolution mass spectra (HRMS) of the related compounds (Fig. S26–S35†), CV and UV measurements, NMR titration, and theoretical calculation details are available in the ESI.†
