# Supplementary material for: π-extended pyrenes: from an antiaromatic buckybowl to doubly curved nanocarbons with gulf architectures
Source: Chem Sci. 2024 Sep 16;15(40):16529–35. doi: 10.1039/d4sc03460k (PMC11421037; doi:10.1039/d4sc03460k)
Supplement: SC-015-D4SC03460K-s001 [file SC-015-D4SC03460K-s001.pdf]

Supplementary Information (SI) for Chemical Science.
This journal is © The Royal Society of Chemistry 2024

Supporting Information for:

π-Extended Pyrenes: From Antiaromatic Buckybowl to Doubly Curved Nanocarbons with Gulf Architectures

Binbin Liu[1†], Zhengxiong Jin[1†], Xinyue Liu[1], Lanfei Sun[3], Cao Yang[2*], and Lei Zhang[1*]

[1] Beijing Advanced Innovation Center for Soft Matter Science and Engineering, State Key Lab of Organic-Inorganic Composites, Beijing University of Chemical Technology, Beijing 100029, P. R. China

[2] School of Materials Science and Engineering, The Key Laboratory of Material Processing and Mold of Ministry of Education, Henan Key Laboratory of Advanced Nylon Materials and Application, Zhengzhou University, Zhengzhou 450001, P. R. China

[3] Shandong North Modern Chemistry Industry Co., Ltd., Jinan 252300, P. R. China

[†] B. Liu and Z. Jin contributed equally to this work.

Table of Contents

1. Synthesis and Characterization of Compounds....S2
2. UV, CV and CD Spectra of Compounds....S3
3. Antiaromaticity of Compound **1**....S3
4. Density Functional Theory Results....S4
5. Chiral HPLC Analysis and Thermal Isomerization of $\mathbf{2}_{meso}$ and $\mathbf{2}_{rac}$....S17
6. Binding Behaviors....S19
7. X-ray Crystallographic Data for Compounds....S24
8. ^{1}H NMR, ^{13}C NMR, and HRMS Spectra of Compounds....S26
9. References....S30

1. Synthesis and Characterization of Compounds

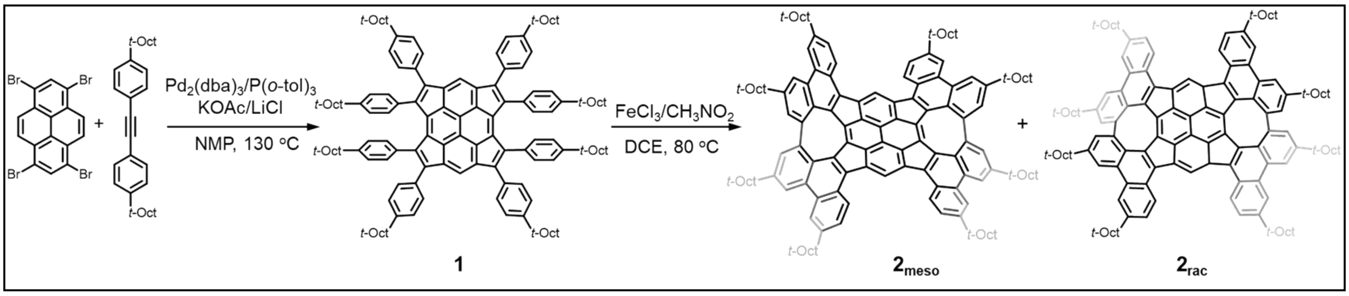

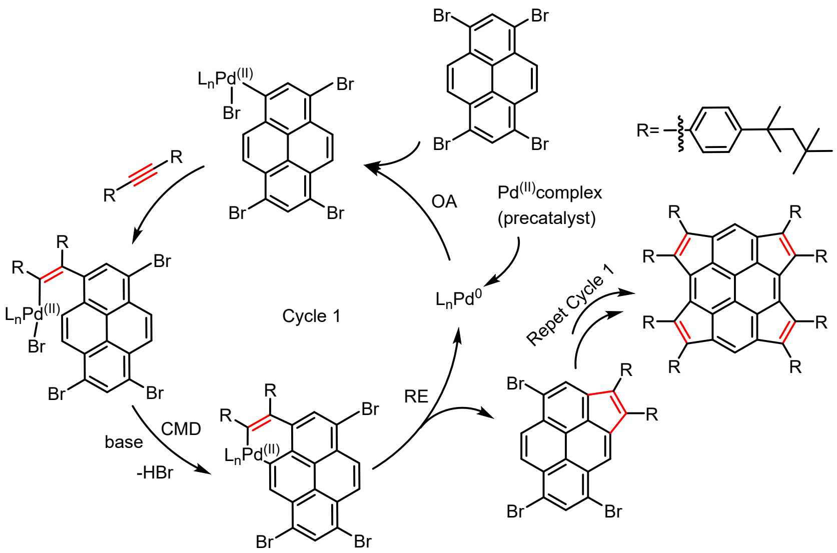

Fig. S1 Proposed mechanism of the palladium-catalyzed cyclopentannulation[1]. (The MS spectrum of two intermediates is provided in Fig. S35)

Compound 1,3,6,8-tetrabromopyrene[2] and 1,2-bis(4-(*tert*-octyl)phenyl)ethyne[3] were prepared according a literature procedures.

Synthesis of Compound 1 In a sealed tube, a solution of 1,3,6,8-tetrabromopyrene (50 mg, 0.097 mmol), 1,2-bis(4-(*tert*-octyl)phenyl)ethyne (156 mg, 0.386 mmol), Pd$_2$(dba)$_3$ (35.5 mg, 0.0386 mmol), P(*o*-Tol)$_3$ (17.7 mg, 0.0582 mmol), KOAc (93mg, 0.97 mmol), LiCl (16.45 mg, 0.388 mmol)and NMP (5 ml) was degassed with nitrogen for 3 times and then stirred at 130 °C for 36 h. After cooling the mixture was to room temperature, the mixture was diluted with dichloromethane and washed with water, and dried over anhydrous sodium sulfate. The organic layer was evaporated and the solid was washed with acetone. The crude product was further purified by short column chromatography on silica gel eluted with dichloromethane and washed with acetone. The product **1** was obtained as yellow solid (35 mg, 20%).

^{1}H NMR (400 MHz, Chloroform-*d*, 298 K) δ 7.08 (d, *J* = 8.3 Hz, 8H), 6.78 (d, *J* = 8.2 Hz, 8H), 6.69 (d, *J* = 8.2 Hz, 8H), 6.61 (d, *J* = 8.4 Hz, 8H), 6.46 (s, 2H), 1.64 (s, 8H), 1.45 (s, 8H), 1.28 (s, 24H), 1.15(s, 24H), 0.69 (s, 36H), 0.63 (s, 36H). ^{13}C NMR (101 MHz, Chloroform-*d*, 298K) δ 148.51, 147.34, 146.80, 144.26, 143.27, 141.78, 138.65, 138.49, 132.22, 132.04, 129.26, 128.30, 125.75, 124.61, 122.16, 117.72, 57.95, 57.28, 38.36, 38.10, 32.39, 32.30, 31.76, 31.74, 31.31, 30.82. HR-MALDI-TOF (m/z): Calcd (%) for $C_{136}H_{170}$: 1803.32033, found: 1803.32988.

Synthesis of Compound 2$_{meso}$ and 2$_{rac}$. In a 100mL two-necked round-bottom flask, **1** (100 mg, 0.055 mmol) was dissolved in dry 1,2-dichloroethane (60 ml) and degassed with nitrogen for 10 min. The mixture was heated to 80°C

and $FeCl_3$ (162 mg, 0.997mmol) in CH_3NO_2 (2 ml) was added dropwise. After 1.5 h, the mixture was quenched with MeOH (15 mL) and was extracted with DCM (30 mL). The organic layer was dried over anhydrous sodium sulfate and the organic layer was evaporated. The crude product was purified by column chromatography on silica gel eluted with dichloromethane: petroleum ether (1 : 20) and further by HPLC (dichloromethane/petroleum ether 9 : 1) to afford compounds $\mathbf{2_{meso}}$ (2 mg, 2%).and $\mathbf{2_{rac}}$ (1.5 mg, 1.5%) as red solid.

$\mathbf{2_{meso}}$: ^{1}H NMR (400 MHz, Chloroform-*d*, 298 K) δ 8.59 (s, 4H), 8.47 (d, *J* = 8.8 Hz, 8H), 8.22 (s, 2H), 7.91 – 7.82 (m, 4H), 6.99 (d, *J* = 1.8 Hz, 4H), 2.07 (d, *J* = 14.9 Hz, 4H), 1.96 (d, *J* = 14.7 Hz, 4H), 1.87 (d, *J* = 14.6 Hz, 4H), 1.75 (d, *J* = 14.6 Hz, 4H), 1.63 (d, *J* = 13.8 Hz, 24H), 1.44 (d, *J* = 7.4 Hz, 24H), 0.86 (s, 36H), 0.81 (s, 36H). ^{13}C NMR (101 MHz, Chloroform-*d*, 298K) δ 149.21, 146.36, 141.48, 141.07, 139.56, 138.97, 136.46, 135.02, 134.44, 132.09, 130.33, 128.62, 127.38, 126.41, 124.37, 121.05, 120.64, 120.30, 119.85, 56.77, 56.64, 39.35, 38.75, 32.59, 32.53, 32.35, 32.08, 32.04, 31.03, 30.23, 29.71. HR-MALDI-TOF (m/z): Calcd (%) for $C_{136}H_{158}$: 1791.23636, found: 1791.23541.

$\mathbf{2_{rac}}$: ^{1}H NMR (400 MHz, Chloroform-*d*, 298 K) δ 8.62 (s, 4H), 8.50 (d, *J* = 9.0 Hz, 8H), 8.31 (s, 2H), 7.93 – 7.86 (m, 4H), 6.98 (d, *J* = 1.7 Hz, 4H), 2.07 (d, *J* = 14.7 Hz, 4H), 1.99 (d, *J* = 14.7 Hz, 4H), 1.87 (d, *J* = 14.6 Hz, 4H), 1.75 (d, *J* = 14.6 Hz, 4H, 1.64 (d, *J* = 7.3 Hz, 24H), 1.45 (d, *J* = 10.5 Hz, 24H), 0.88 (s, 36H), 0.80 (s, 36H). ^{13}C NMR (100 MHz, Chloroform-*d*, 298K) δ 149.16, 146.30, 141.71, 141.11, 139.65, 139.26, 136.63, 135.04, 134.37, 132.09, 130.37, 128.63, 127.50, 126.47, 124.36, 121.02, 120.29, 119.60, 56.81, 56.64, 39.35, 38.74, 32.60, 32.52, 32.21, 32.08, 32.03, 31.20, 30.23, 29.71. HR-MALDI-TOF (m/z): Calcd (%) for $C_{136}H_{158}$: 1791.23636, found: 1791.23597.

2. UV, CV and CD Spectra of Compounds

UV-vis absorption spectra were measured with Shimadzu (UV- 3600i Plus) in $CHCl_3$ at room temperature; Circular dichroism (CD) measurements were carried out on a J-815-150S Spectrometer in $CHCl_3$ at room temperature. Cyclic voltammetry (CV) was recorded on a CHI620E electrochemical workstation using glassy carbon discs as the working electrode, Pt wire as the counter electrode, Ag/Ag^+ electrode as the reference electrode, and ferrocene/ferrocenium as an internal potential marker. The experiments were performed in nitrogen-purged DCM with tetrabutylammonium hexafluorophosphate ($TBAPF_6$, 0.1 M) as the supporting electrolyte with a scan of 100 mV/s.

3. Antiaromaticity of Compound 1

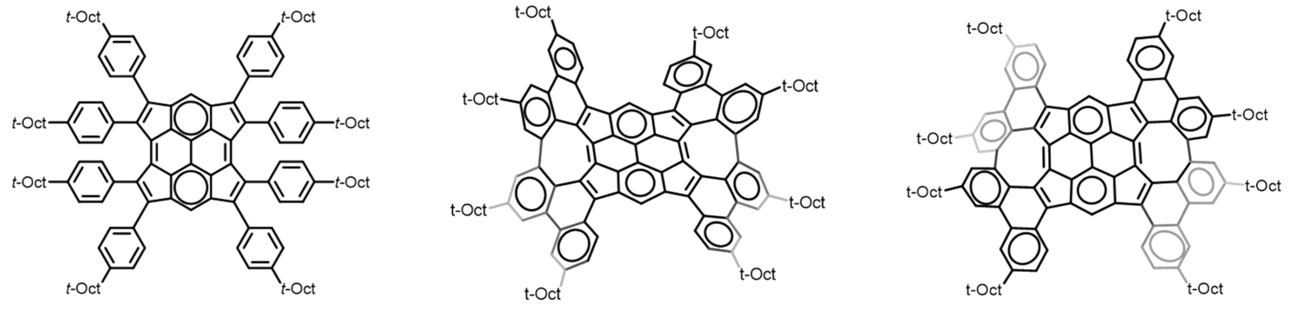

Fig. S2 The most representative Clar structures of **1**, $\mathbf{2_{rac}}$, and $\mathbf{2_{meso}}$.

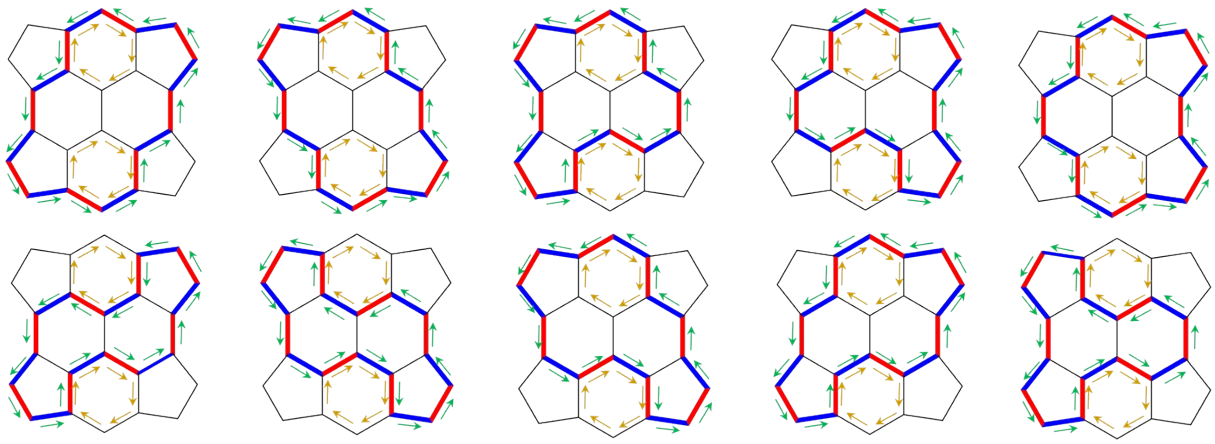

Fig. S3 The possible resonance structures for TPP with two Clar's sextets and total 4n 16 π-electrons. The conjugated pathways (in bold) with an alternation of the C-C single (red) and double (blue) bonds. Green arrow: paratropic current; yellow arrow: diatropic current.

4. Density Functional Theory Results

All density functional theory (DFT) calculations were performed using Gaussian 09, Revision D.01 program.[4] All optimized structures were conducted at the B3LYP level with the 6-311G(d,p) basis set at gas phase, the same level of frequency analysis confirmed that all optimized structures were energy minima structures on the potential energy surface. Time-dependent density functional theory (TD-DFT) and were calculated at the B3LYP/6-311G(d,p) level in $CHCl_3$ with the IEFPCM solvent model and at least the 100 excited states were considered. For better comparison with experimental data, Lorentz broadening with a half width at half height of 0.3 eV was applied to obtain the line spectra. CD spectra and Major orbital transition contributions in excited states were generated using Multiwfn 3.8 software.[5] Independent gradient model based on Hirshfeld partition (IGMH) method[6] was performed in Multiwfn and the visual isosurface images were obtained by VMD.[7] NICS values were calculated at the B3LYP/6-311G(d,p) using the standard GIAO procedure;[8] ACID plot was calculated by using the method developed by Herges based on the optimized ground-state geometries.[9] The optimization and frequency calculations of the transition states (TS) were conducted at the B3LYP/6-311G(d,p) level at gas phase and only one imaginary frequency exists. The connection between the transition states and the ground state was confirmed by IRC calculation.

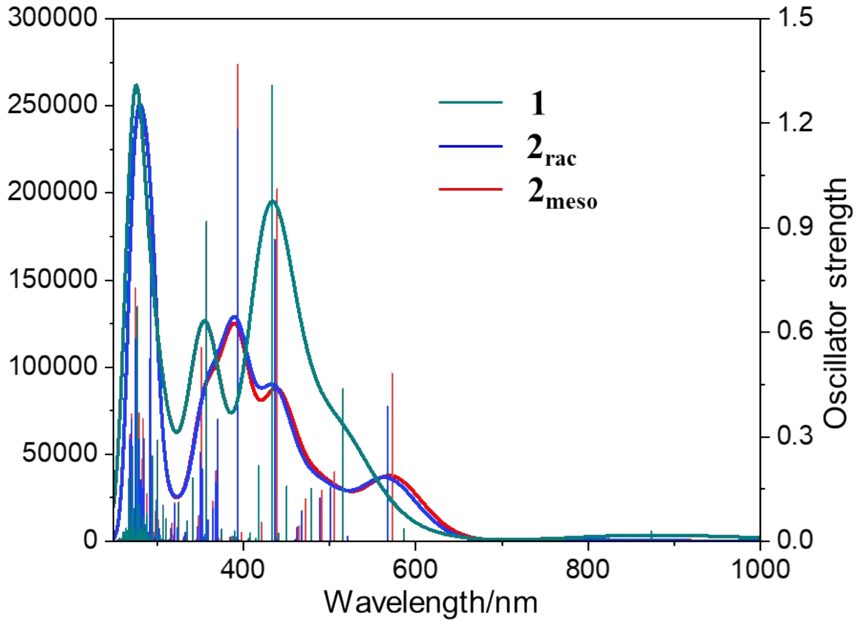

Fig. S4 Calculated UV-vis spectra of **1**, $\mathbf{2_{meso}}$ and $\mathbf{2_{rac}}$ at the B3LYP/6-311G(d,p) level in $CHCl_3$ solution.

Table S1. TD-DFT singlet excitation energies E, excitation wavelengths λ, oscillator strengths *f*, and orbital contributions of **1** at the B3LYP/6-311G(d,p) level in $CHCl_3$ solution.

Excited state	Energy (eV)	Wavelength (nm)	*f*	Contributions
S_1	1.2925	959.26	0.00010	HOMO→LUMO: 99.3%
S_5	2.4044	515.66	0.36470	HOMO-4→LUMO: 85.7%<br>HOMO→LUMO+1: 12.1%
S_8	2.5894	478.81	0.12480	HOMO-6→LUMO: 87.3%
S_{10}	2.7525	450.44	0.13160	HOMO-1→LUMO+1: 89.1%<br>HOMO-6→LUMO: 5.8%
S_{13}	2.8617	433.25	1.08960	HOMO→LUMO+1: 76.3%<br>HOMO-4→LUMO: 9.3%<br>HOMO-11→LUMO: 9.2%
S_{15}	2.9688	417.62	0.18110	HOMO-11→LUMO: 82.6%<br>HOMO-12→LUMO: 7.7%<br>HOMO→LUMO+1: 6.7%

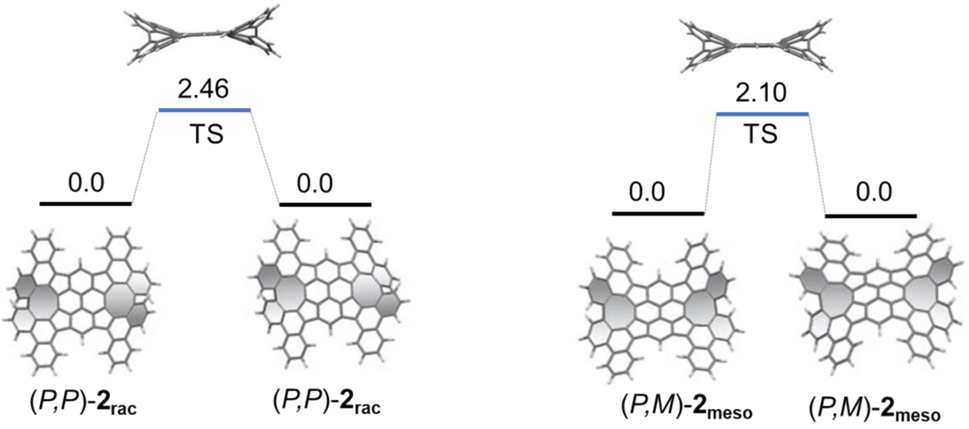

Fig. S5 Calculated diagram for the bowl inversion process of (*P*,*P*)-$\mathbf{2}_{\mathbf{rac}}$ (left) and (*P*,*M*)-$\mathbf{2}_{\mathbf{meso}}$ (right)

Table S2. Cartesian coordinates of optimized structures ((*P*,*P*)-$\mathbf{2}_{\mathbf{rac}}$) calculated by B3LYP/6-311G(d,p) level of theory.

(*P*,*P*)-$\mathbf{2}_{\mathbf{rac}}$							
C	-1.04876000	1.48223900	-0.70981200	H	-3.99287500	7.91125500	1.86786300
C	0.07092900	0.69242400	-0.85297900	H	-7.35696000	2.16095200	3.16919500
C	1.32923600	1.21673300	-0.67571800	H	-8.42940700	-0.74391500	-2.26293200
C	1.52121500	2.58171500	-0.43370900	H	-6.08296900	-7.00302100	-0.90775400
C	0.37622800	3.40469300	-0.29137300	C	1.04876700	-1.48230300	-0.70964000
H	0.48378500	4.43882100	-0.00199600	C	-0.07091900	-0.69250200	-0.85290500
C	-0.92338200	2.84225700	-0.41150700	C	-1.32922900	-1.21679300	-0.67561900
C	-2.30047300	3.25711900	-0.04100000	C	-1.52121300	-2.58175400	-0.43348900
C	-2.73900300	4.52496200	0.44021700	C	-0.37623000	-3.40471800	-0.29105200
C	-2.00226300	5.71322900	0.23509400	H	-0.48379400	-4.43882000	-0.00158500
H	-1.10637400	5.67857300	-0.36893300	C	0.92338400	-2.84229400	-0.41121200
C	-2.43972500	6.91547500	0.74484000	C	2.30047100	-3.25712300	-0.04065200
H	-1.87299100	7.82167000	0.56650100	C	2.73899900	-4.52492600	0.44067300
C	-3.63662600	6.96562600	1.47652800	C	2.00226800	-5.71321300	0.23563600
C	-4.37906400	5.81955900	1.67530800	H	1.10638600	-5.67861200	-0.36840400

H	-5.32016100	5.89778100	2.20198900	C	2.43973000	-6.91541700	0.74548300
C	-3.96047600	4.57186400	1.16632600	H	1.87300200	-7.82162900	0.56721000
C	-4.74811600	3.35912800	1.33858400	C	3.63662400	-6.96550400	1.47718700
C	-5.80042900	3.32527900	2.27202700	C	4.37905400	-5.81941800	1.67588400
H	-5.97777800	4.17897900	2.91077100	H	5.32014700	-5.89759200	2.20257900
C	-6.57820000	2.20101800	2.41716400	C	3.96046400	-4.57176500	1.16680000
C	-6.39771400	1.12946900	1.53950000	C	4.74809600	-3.35901300	1.33897200
H	-7.08308600	0.29231100	1.57929300	C	5.80039600	-3.32508700	2.27242700
C	-5.39134000	1.11141000	0.57618800	H	5.97773700	-4.17873600	2.91124100
C	-5.64354100	0.02282100	-0.44472300	C	6.57816400	-2.20081300	2.41748400
C	-6.85714200	0.17146200	-1.10553500	C	6.39768800	-1.12933600	1.53972900
H	-7.34838900	1.13627500	-1.07160600	H	7.08306000	-0.29217500	1.57946100
C	-7.49614300	-0.90361100	-1.73639400	C	5.39132700	-1.11135600	0.57640200
C	-6.97743400	-2.17165600	-1.60571600	C	5.64354700	-0.02285500	-0.44459800
H	-7.51699400	-3.01139600	-2.02127800	C	6.85716400	-0.17155200	-1.10536900
C	-5.73039300	-2.37733000	-0.98239100	H	7.34841200	-1.13636100	-1.07134300
C	-5.17987300	-3.71375900	-0.80418000	C	7.49617800	0.90346700	-1.73630800
C	-5.96728600	-4.87109500	-0.97650900	C	6.97746200	2.17152200	-1.60575400
H	-7.01318600	-4.77397400	-1.23397900	H	7.51703000	3.01122700	-2.02137800
C	-5.44737400	-6.13436300	-0.78245500	C	5.73040800	2.37724800	-0.98247600
C	-4.10468700	-6.29518900	-0.40683600	C	5.17988200	3.71369200	-0.80439500
H	-3.70033800	-7.28750400	-0.24569500	C	5.96729900	4.87101300	-0.97680100
C	-3.30656300	-5.18653200	-0.23290500	H	7.01320700	4.77387000	-1.23423400
H	-2.27913300	-5.31203300	0.07561400	C	5.44738200	6.13429700	-0.78286800
C	-3.81565200	-3.88234700	-0.42200000	C	4.10468600	6.29515500	-0.40729700
C	-3.00397900	-2.71230000	-0.32113000	H	3.70033300	7.28748400	-0.24624900
C	-3.58532900	-1.43461300	-0.32790000	C	3.30655700	5.18651300	-0.23329300
C	-2.52978700	-0.42059100	-0.50009100	H	2.27911900	5.31204000	0.07519200
C	-2.38826000	0.96001800	-0.49445400	C	3.81565100	3.88231200	-0.42226500
C	-3.16017200	2.14420700	-0.06540500	C	3.00397600	2.71227300	-0.32131800
C	-4.44840200	2.18613900	0.56665900	C	3.58532800	1.43458600	-0.32795600
C	-4.98643200	-1.24209500	-0.53598900	C	2.52979100	0.42054600	-0.50008500
H	6.08298100	7.00294500	-0.90822400	C	2.38826400	-0.96006200	-0.49430900
H	8.42945400	0.74372500	-2.26281100	C	3.16016800	-2.14421100	-0.06513800
H	7.35691300	-2.16068500	3.16952100	C	4.44838900	-2.18608700	0.56694800
H	3.99287500	-7.91110000	1.86859900	C	4.98643700	1.24205100	-0.53599200

TS

C	2.48567800	0.69526900	0.00391100	C	-3.36930000	6.69197500	0.55198100
C	2.48554000	-0.69579500	-0.00495600	C	-2.72927800	5.51938300	0.21828400
C	1.19478000	-1.35441800	-0.02607400	C	-5.58832700	0.56518600	0.50697600
C	-0.00007100	-0.69109200	-0.00089800	C	-6.71063700	0.51750800	1.32811500
C	0.00007100	0.69109200	-0.00090100	C	-7.13999900	1.62000000	2.07499400
C	1.19505300	1.35416400	0.02459700	C	-6.50071100	2.82852500	1.92494400
C	-1.19478000	1.35441800	-0.02608000	C	-5.33581300	2.93324200	1.14071000
C	-1.23785500	2.74856800	0.00390700	H	5.17589900	-7.56780000	1.34860400
C	0.00036600	3.45735400	-0.00042800	H	8.00589600	-1.53163000	2.72007300
C	1.23844200	2.74831400	-0.00492700	H	8.00751300	1.53018000	-2.71833000
C	-1.19505400	-1.35416400	0.02460100	H	5.17794600	7.56680800	-1.34808300

C	-2.48567800	-0.69526900	0.00391200	H	-5.17589900	7.56780500	1.34857400
C	-2.48554000	0.69579500	-0.00496100	H	-8.00589800	1.53164000	2.72006000
C	1.23785500	-2.74856800	0.00391800	C	-2.70167700	-3.03120400	-0.12955300
C	-0.00036600	-3.45735400	-0.00041500	C	-3.42569800	-1.82180600	-0.11807600
C	-1.23844200	-2.74831400	-0.00491800	C	-4.79308400	-1.75348300	-0.53573700
C	3.42569800	1.82180600	-0.11807900	C	-3.35406800	-4.26613800	-0.41459000
C	2.70167700	3.03120400	-0.12956100	C	-2.73055800	-5.51884500	-0.21860600
C	-2.70098200	3.03175100	0.12902400	C	-3.37092200	-6.69132000	-0.55205500
C	-3.42527500	1.82251200	0.11761700	C	-4.66403800	-6.64652700	-1.09659700
C	3.42527500	-1.82251100	0.11762700	C	-5.29476400	-5.43487000	-1.29215300
C	2.70098200	-3.03175000	0.12903800	C	-4.66963700	-4.21456700	-0.96019600
C	3.35406900	4.26613700	-0.41460300	C	-5.33690400	-2.93222000	-1.14024000
C	4.79308400	1.75348100	-0.53573900	C	-6.50216000	-2.82731400	-1.92391900
C	-3.35299300	4.26680000	0.41441000	C	-7.14130500	-1.61867900	-2.07369500
C	-4.79249300	1.75443100	0.53589000	C	-6.71138700	-0.51623600	-1.32705900
C	3.35299300	-4.26679900	0.41442900	C	-5.58868100	-0.56408900	-0.50647700
C	4.79249200	-1.75442900	0.53590000	H	-5.17794500	-7.56681300	-1.34806000
C	5.33690500	2.93221600	-1.14024600	H	-8.00751000	-1.53019000	-2.71833100
C	6.50216100	2.82730700	-1.92392400	H	0.00046200	4.53696000	-0.00020000
C	7.14130700	1.61867200	-2.07369400	H	-0.00046200	-4.53696000	-0.00018300
C	6.71138900	0.51623100	-1.32705500	H	6.87774800	3.69493000	-2.44818200
C	5.58868100	0.56408700	-0.50647400	H	7.29694600	-0.39461100	-1.34361000
C	5.58832700	-0.56518400	0.50698300	H	7.29633600	0.39324300	1.34494800
C	6.71063600	-0.51750300	1.32812300	H	6.87586900	-3.69619400	2.44943700
C	7.13999700	-1.61999300	2.07500600	H	6.30391000	-5.43416900	1.67721200
C	6.50070900	-2.82851800	1.92495900	H	2.88334900	-7.64549600	0.38232500
C	5.33581300	-2.93323700	1.14072500	H	1.74879400	-5.55238100	-0.23511100
C	4.66838200	-4.21546800	0.96047300	H	1.74993700	5.55203600	0.23447800
C	5.29316200	-5.43588100	1.29267500	H	2.88510700	7.64493200	-0.38251700
C	4.66225500	-6.64741800	1.09695300	H	6.30563500	5.43296800	-1.67638100
C	3.36929900	-6.69197300	0.55200800	H	-6.30391100	5.43417500	1.67718800
C	2.72927800	-5.51938200	0.21830700	H	-2.88334900	7.64549700	0.38229500
C	2.73055900	5.51884400	-0.21862300	H	-1.74879400	5.55238000	-0.23513400
C	3.37092200	6.69131800	-0.55207600	H	-7.29633700	-0.39323800	1.34494300
C	4.66403800	6.64652300	-1.09661700	H	-6.87587000	3.69620200	2.44941800
C	5.29476500	5.43486500	-1.29216800	H	-1.74993700	-5.55203500	0.23449600
C	4.66963700	4.21456400	-0.96020700	H	-2.88510700	-7.64493400	-0.38249200
C	-4.66838300	4.21547200	0.96045400	H	-6.30563400	-5.43297400	-1.67636700
C	-5.29316200	5.43588600	1.29265200	H	-6.87774600	-3.69493900	-2.44817500
C	-4.66225500	6.64742200	1.09692600	H	-7.29694500	0.39460700	-1.34361800

Table S3. Cartesian coordinates of optimized structures ((*P*,*M*)-**2**$_{\mathbf{meso}}$) calculated by B3LYP/6-311G(d,p) level of theory.

(*P*,*M*)-**2**$_{\mathbf{meso}}$							
C	-1.20282800	1.25958400	-0.93256100	H	-8.25536800	-1.98397400	-2.02798800

C	0.00001300	0.58548000	-0.94137800	H	-5.27532300	-7.62370100	0.39590600
C	1.20282500	1.25962800	-0.93256900	H	4.83839700	7.69450500	0.49645000
C	1.23037600	2.65764000	-0.89291500	C	1.18990100	-1.37922000	-0.29828300
C	-0.00004300	3.36438700	-0.86453200	C	0.00004100	-0.75589700	-0.57316100
H	-0.00006800	4.43114400	-0.69641600	C	-1.18979500	-1.37926200	-0.29827700
C	-1.23044400	2.65758900	-0.89293600	C	-1.23106200	-2.70700400	0.13804300
C	-2.64636500	2.98266200	-0.57962000	C	0.00009800	-3.39033200	0.33529400
C	-3.22144500	4.26135900	-0.32465600	H	0.00010100	-4.42174000	0.65327200
C	-2.61072800	5.46599000	-0.74017800	C	1.23122200	-2.70697200	0.13802600
H	-1.70731000	5.41957200	-1.33208100	C	2.69558300	-2.98170400	0.25367300
C	-3.17718800	6.68619500	-0.44596600	C	3.37489800	-4.23315900	0.35081000
H	-2.70565000	7.60164400	-0.78320300	C	2.73579700	-5.41806800	0.77855800
C	-4.38118100	6.73752400	0.27387700	H	1.71444400	-5.36772000	1.12734800
C	-5.00142400	5.57254600	0.67688600	C	3.40362800	-6.62235100	0.79324700
H	-5.95073100	5.64305100	1.18968900	H	2.90154200	-7.51935300	1.13591100
C	-4.44878500	4.30580300	0.39247200	C	4.74208900	-6.68061200	0.37488900
C	-5.10577100	3.06620000	0.78429700	C	5.38954800	-5.53608900	-0.04357400
C	-6.16217000	3.08691300	1.71376800	H	6.43145800	-5.60290900	-0.32578000
H	-6.44080400	4.01718900	2.18826700	C	4.73817500	-4.28543000	-0.06727100
C	-6.81600200	1.92934000	2.06390300	C	5.42584500	-3.06825000	-0.47620100
C	-6.50768000	0.74224500	1.39507700	C	6.67254100	-3.11001200	-1.13164000
H	-7.09956400	-0.14328200	1.58940600	H	7.10882200	-4.06229200	-1.39975100
C	-5.49121000	0.65994900	0.44618800	C	7.32215000	-1.94741100	-1.47915700
C	-5.60900600	-0.61541200	-0.36159200	C	6.81650700	-0.71623000	-1.04222300
C	-6.81648600	-0.71655000	-1.04209500	H	7.40820700	0.18058300	-1.18001000
H	-7.40825300	0.18022500	-1.17984900	C	5.60907800	-0.61518700	-0.36160800
C	-7.32209700	-1.94777200	-1.47895400	C	5.49123100	0.66016500	0.44621200
C	-6.67240200	-3.11032800	-1.13144500	C	6.50756400	0.74243500	1.39525200
H	-7.10864100	-4.06264700	-1.39949100	H	7.09943800	-0.14308300	1.58963200
C	-5.42568600	-3.06847100	-0.47605700	C	6.81576200	1.92950900	2.06417800
C	-4.73795700	-4.28560300	-0.06707600	C	6.16194000	3.08707800	1.71401400
C	-5.38930400	-5.53626800	-0.04326300	H	6.44046800	4.01733200	2.18861600
H	-6.43122300	-5.60312500	-0.32542300	C	5.10563200	3.06637300	0.78443600
C	-4.74180000	-6.68074700	0.37525800	C	4.44858100	4.30596500	0.39266500
C	-3.40332600	-6.62242400	0.79355800	C	5.00112700	5.57272700	0.67716300
H	-2.90120500	-7.51938800	1.13627100	H	5.95042400	5.64326800	1.18997400
C	-2.73552000	-5.41812400	0.77875800	C	4.38079700	6.73768700	0.27422900
H	-1.71415500	-5.36772800	1.12750400	C	3.17679700	6.68631500	-0.44560000
C	-3.37466600	-4.23326700	0.35095100	H	2.70517800	7.60175100	-0.78275800
C	-2.69540100	-2.98179000	0.25372800	C	2.61042300	5.46608700	-0.73988600
C	-3.41417400	-1.79553700	0.02720500	H	1.70698100	5.41964600	-1.33175300
C	-2.47338600	-0.71253800	-0.30550800	C	3.22124900	4.26147800	-0.32447300
C	-2.47815100	0.64701500	-0.58770400	C	2.64626300	2.98275900	-0.57951800
C	-3.37943400	1.79833500	-0.38931800	C	3.37940000	1.79846600	-0.38928300

C	-4.67164000	1.81280800	0.23570300	C	2.47817000	0.64711200	-0.58770200
C	-4.82070600	-1.79863400	-0.22536900	C	2.47345600	-0.71244700	-0.30550300
H	-4.83885300	7.69432000	0.49604100	C	3.41429900	-1.79541000	0.02716600
H	5.27563400	-7.62355500	0.39544500	C	4.82082500	-1.79844700	-0.22543100
H	8.25538700	-1.98355100	-2.02825300	C	4.67160500	1.81299100	0.23574100
H	-7.59748900	1.94169100	2.81408900	H	7.59713800	1.94184500	2.81448000
				**TS**			
C	-1.19539500	1.31291300	-0.33771200	H	-7.98348900	-1.97462300	-2.43187300
C	0.00000000	0.66242500	-0.19987300	H	-5.14210000	-7.68847500	-0.06139800
C	1.19539500	1.31291300	-0.33771200	H	5.14191600	7.68850300	0.06176000
C	1.23771100	2.69460900	-0.53213600	C	1.19538900	-1.31296200	0.33720400
C	0.00000000	3.39904000	-0.61178400	C	0.00000000	-0.66249600	0.19924700
H	0.00000000	4.47702100	-0.67816600	C	-1.19539000	-1.31296200	0.33720400
C	-1.23771100	2.69461000	-0.53213600	C	-1.23771200	-2.69463100	0.53182100
C	-2.69735900	3.00108800	-0.42463800	C	0.00000000	-3.39905400	0.61155500
C	-3.34256200	4.26901200	-0.33760700	H	0.00000000	-4.47702300	0.67813100
C	-2.71918000	5.46813100	-0.74994200	C	1.23771100	-2.69463100	0.53182100
H	-1.74579300	5.41884300	-1.21734300	C	2.69737900	-3.00108900	0.42449500
C	-3.35066100	6.68325800	-0.60604000	C	3.34262700	-4.26900100	0.33761100
H	-2.86506000	7.59287700	-0.93908100	C	2.71923400	-5.46811300	0.74995000
C	-4.63490100	6.73639000	-0.04163700	H	1.74579600	-5.41882400	1.21724700
C	-5.26634100	5.57741400	0.36143600	C	3.35075800	-6.68323400	0.60617800
H	-6.27098300	5.64531800	0.75583200	H	2.86514400	-7.59284600	0.93921900
C	-4.65029900	4.31540800	0.22768800	C	4.63505400	-6.73636700	0.04190400
C	-5.31906800	3.08385200	0.62625200	C	5.26650600	-5.57739800	-0.36117300
C	-6.47896000	3.11494900	1.42426300	H	6.27118700	-5.64530000	-0.75547000
H	-6.84992300	4.05909400	1.79797600	C	4.65042200	-4.31539900	-0.22755500
C	-7.12025900	1.95045400	1.77763100	C	5.31920500	-3.08385200	-0.62612100
C	-6.69712800	0.73639200	1.22544700	C	6.47918200	-3.11495600	-1.42401000
H	-7.28379000	-0.15726300	1.39892700	H	6.85021400	-4.05910800	-1.79763500
C	-5.57893600	0.64278000	0.40290700	C	7.12048400	-1.95045800	-1.77736900
C	-5.57898800	-0.64276000	-0.40286500	C	6.69726700	-0.73638000	-1.22528300
C	-6.69726700	-0.73637900	-1.22528400	H	7.28393200	0.15727900	-1.39873400
H	-7.28393100	0.15728000	-1.39873600	C	5.57898800	-0.64276100	-0.40286500
C	-7.12048400	-1.95045800	-1.77737000	C	5.57893600	0.64277900	0.40290700
C	-6.47918200	-3.11495500	-1.42401100	C	6.69712700	0.73639100	1.22544600
H	-6.85021500	-4.05910800	-1.79763600	H	7.28379000	-0.15726400	1.39892700
C	-5.31920600	-3.08385200	-0.62612200	C	7.12026000	1.95045300	1.77763000
C	-4.65042300	-4.31539900	-0.22755600	C	6.47896100	3.11494900	1.42426200
C	-5.26650600	-5.57739800	-0.36117500	H	6.84992500	4.05909300	1.79797300
H	-6.27118800	-5.64530000	-0.75547200	C	5.31906800	3.08385200	0.62625000
C	-4.63505400	-6.73636700	0.04190100	C	4.65030000	4.31540800	0.22768600
C	-3.35075700	-6.68323400	0.60617500	C	5.26634200	5.57741300	0.36143300
H	-2.86514400	-7.59284600	0.93921500	H	6.27098500	5.64531700	0.75582800

C	-2.71923300	-5.46811300	0.74994700	C	4.63490200	6.73639000	-0.04164000
H	-1.74579600	-5.41882400	1.21724400	C	3.35066200	6.68325800	-0.60604300
C	-3.34262800	-4.26900100	0.33761000	H	2.86506100	7.59287700	-0.93908300
C	-2.69738000	-3.00108900	0.42449400	C	2.71918000	5.46813000	-0.74994400
C	-3.42235500	-1.81053600	0.21836500	H	1.74579300	5.41884300	-1.21734400
C	-2.48454300	-0.67917800	0.15000400	C	3.34256300	4.26901100	-0.33760800
C	-2.48454500	0.67915100	-0.15037400	C	2.69735900	3.00108700	-0.42463800
C	-3.42234000	1.81053700	-0.21852100	C	3.42234000	1.81053700	-0.21852100
C	-4.78359100	1.81854500	0.22337900	C	2.48454500	0.67915100	-0.15037500
C	-4.78365500	-1.81853800	-0.22337500	C	2.48454300	-0.67917800	0.15000400
H	-5.14191400	7.68850400	0.06176400	C	3.42235400	-1.81053600	0.21836500
H	5.14210000	-7.68847500	-0.06139500	C	4.78365400	-1.81853800	-0.22337500
H	7.98348900	-1.97462400	-2.43187100	C	4.78359100	1.81854500	0.22337900
H	-7.98319500	1.97461100	2.43222500	H	7.98319600	1.97461000	2.43222300

Table S4. Cartesian coordinates of optimized structures ((*P*,*P*)-$\mathbf{2}_{\mathbf{meso}}$, (*P*,*M*)-$\mathbf{2}_{\mathbf{meso}}$ and (*M*,*M*)-$\mathbf{2}_{\mathbf{meso}}$) calculated by B3LYP/6-311G(d,p) level of theory.

				(*P*,*P*)-$\mathbf{2}_{\mathbf{rac}}$			
C	-1.04876000	1.48223900	-0.70981200	H	-3.99287500	7.91125500	1.86786300
C	0.07092900	0.69242400	-0.85297900	H	-7.35696000	2.16095200	3.16919500
C	1.32923600	1.21673300	-0.67571800	H	-8.42940700	-0.74391500	-2.26293200
C	1.52121500	2.58171500	-0.43370900	H	-6.08296900	-7.00302100	-0.90775400
C	0.37622800	3.40469300	-0.29137300	C	1.04876700	-1.48230300	-0.70964000
H	0.48378500	4.43882100	-0.00199600	C	-0.07091900	-0.69250200	-0.85290500
C	-0.92338200	2.84225700	-0.41150700	C	-1.32922900	-1.21679300	-0.67561900
C	-2.30047300	3.25711900	-0.04100000	C	-1.52121300	-2.58175400	-0.43348900
C	-2.73900300	4.52496200	0.44021700	C	-0.37623000	-3.40471800	-0.29105200
C	-2.00226300	5.71322900	0.23509400	H	-0.48379400	-4.43882000	-0.00158500
H	-1.10637400	5.67857300	-0.36893300	C	0.92338400	-2.84229400	-0.41121200
C	-2.43972500	6.91547500	0.74484000	C	2.30047100	-3.25712300	-0.04065200
H	-1.87299100	7.82167000	0.56650100	C	2.73899900	-4.52492600	0.44067300
C	-3.63662600	6.96562600	1.47652800	C	2.00226800	-5.71321300	0.23563600
C	-4.37906400	5.81955900	1.67530800	H	1.10638600	-5.67861200	-0.36840400
H	-5.32016100	5.89778100	2.20198900	C	2.43973000	-6.91541700	0.74548300
C	-3.96047600	4.57186400	1.16632600	H	1.87300200	-7.82162900	0.56721000
C	-4.74811600	3.35912800	1.33858400	C	3.63662400	-6.96550400	1.47718700
C	-5.80042900	3.32527900	2.27202700	C	4.37905400	-5.81941800	1.67588400
H	-5.97777800	4.17897900	2.91077100	H	5.32014700	-5.89759200	2.20257900
C	-6.57820000	2.20101800	2.41716400	C	3.96046400	-4.57176500	1.16680000
C	-6.39771400	1.12946900	1.53950000	C	4.74809600	-3.35901300	1.33897200
H	-7.08308600	0.29231100	1.57929300	C	5.80039600	-3.32508700	2.27242700
C	-5.39134000	1.11141000	0.57618800	H	5.97773700	-4.17873600	2.91124100
C	-5.64354100	0.02282100	-0.44472300	C	6.57816400	-2.20081300	2.41748400
C	-6.85714200	0.17146200	-1.10553500	C	6.39768800	-1.12933600	1.53972900
H	-7.34838900	1.13627500	-1.07160600	H	7.08306000	-0.29217500	1.57946100

C	-7.49614300	-0.90361100	-1.73639400	C	5.39132700	-1.11135600	0.57640200
C	-6.97743400	-2.17165600	-1.60571600	C	5.64354700	-0.02285500	-0.44459800
H	-7.51699400	-3.01139600	-2.02127800	C	6.85716400	-0.17155200	-1.10536900
C	-5.73039300	-2.37733000	-0.98239100	H	7.34841200	-1.13636100	-1.07134300
C	-5.17987300	-3.71375900	-0.80418000	C	7.49617800	0.90346700	-1.73630800
C	-5.96728600	-4.87109500	-0.97650900	C	6.97746200	2.17152200	-1.60575400
H	-7.01318600	-4.77397400	-1.23397900	H	7.51703000	3.01122700	-2.02137800
C	-5.44737400	-6.13436300	-0.78245500	C	5.73040800	2.37724800	-0.98247600
C	-4.10468700	-6.29518900	-0.40683600	C	5.17988200	3.71369200	-0.80439500
H	-3.70033800	-7.28750400	-0.24569500	C	5.96729900	4.87101300	-0.97680100
C	-3.30656300	-5.18653200	-0.23290500	H	7.01320700	4.77387000	-1.23423400
H	-2.27913300	-5.31203300	0.07561400	C	5.44738200	6.13429700	-0.78286800
C	-3.81565200	-3.88234700	-0.42200000	C	4.10468600	6.29515500	-0.40729700
C	-3.00397900	-2.71230000	-0.32113000	H	3.70033300	7.28748400	-0.24624900
C	-3.58532900	-1.43461300	-0.32790000	C	3.30655700	5.18651300	-0.23329300
C	-2.52978700	-0.42059100	-0.50009100	H	2.27911900	5.31204000	0.07519200
C	-2.38826000	0.96001800	-0.49445400	C	3.81565100	3.88231200	-0.42226500
C	-3.16017200	2.14420700	-0.06540500	C	3.00397600	2.71227300	-0.32131800
C	-4.44840200	2.18613900	0.56665900	C	3.58532800	1.43458600	-0.32795600
C	-4.98643200	-1.24209500	-0.53598900	C	2.52979100	0.42054600	-0.50008500
H	6.08298100	7.00294500	-0.90822400	C	2.38826400	-0.96006200	-0.49430900
H	8.42945400	0.74372500	-2.26281100	C	3.16016800	-2.14421100	-0.06513800
H	7.35691300	-2.16068500	3.16952100	C	4.44838900	-2.18608700	0.56694800
H	3.99287500	-7.91110000	1.86859900	C	4.98643700	1.24205100	-0.53599200

TS$_1$

C	-1.29714900	-1.31680900	-0.80376700	H	-5.70929800	-7.31656200	-0.08484600
C	-0.07265300	-0.77006500	-1.12889600	H	-8.41153300	-1.42682200	-2.23778000
C	1.08400000	-1.47209200	-0.83770900	H	-7.46180300	2.07656500	2.85011500
C	1.03127600	-2.69596500	-0.16249300	H	-4.24935200	7.81480400	1.22546100
C	-0.23011600	-3.31272200	0.05137200	C	1.24778200	1.21543000	-1.15319500
H	-0.28598900	-4.27591700	0.53314100	C	0.00551500	0.61915900	-1.31463400
C	-1.40883700	-2.62082300	-0.29168400	C	-1.15163200	1.36426400	-1.14451000
C	-2.87662400	-2.81944200	-0.12616000	C	-1.07803800	2.73155300	-0.83860600
C	-3.62033700	-4.03661100	-0.05122900	C	0.18148000	3.32530600	-0.61356500
C	-3.03549500	-5.27441300	0.29682400	H	0.22960200	4.33919600	-0.25041200
H	-1.99659300	-5.30422200	0.59047100	C	1.35662700	2.54475700	-0.72535900
C	-3.77028000	-6.43915500	0.28360100	C	2.76741700	2.69679000	-0.30159500
H	-3.30646600	-7.37780300	0.56272000	C	3.39788100	3.80266400	0.33826700
C	-5.12371900	-6.40479900	-0.08593100	C	2.70034900	4.88212200	0.92798100
C	-5.71615600	-5.20984600	-0.43920600	H	1.62160600	4.89203600	0.91753900
H	-6.76735200	-5.20588300	-0.69360800	C	3.37575000	5.89042400	1.57945400
C	-4.99463100	-3.99816500	-0.43479300	H	2.82375800	6.69865300	2.04451000
C	-5.62096600	-2.73166200	-0.78682700	C	4.77601200	5.85191000	1.66875000
C	-6.88265900	-2.67615400	-1.41353300	C	5.47634500	4.81038300	1.09597600

H	-7.38122200	-3.59082800	-1.70322200	H	6.55176800	4.77399800	1.20955600
C	-7.46775600	-1.46513500	-1.70715900	C	4.81259900	3.76850000	0.41615900
C	-6.88540800	-0.28256300	-1.23088900	C	5.54245400	2.69451500	-0.24011900
H	-7.43139300	0.64971100	-1.31029800	C	6.83188900	2.98344300	-0.70148100
C	-5.66194800	-0.28118800	-0.57334600	H	7.26674900	3.95616700	-0.52309900
C	-5.46177600	0.91252600	0.33209300	C	7.46686900	2.07211200	-1.50584900
C	-6.47172100	0.96821100	1.29173700	C	6.92557500	0.80207400	-1.65489800
H	-7.12878500	0.11390600	1.39216000	H	7.53590700	0.13581100	-2.23271100
C	-6.68304100	2.08800200	2.09712000	C	5.73159100	0.34803900	-1.06988200
C	-5.92945400	3.21700900	1.88292900	C	5.63881800	-1.17780300	-0.91135200
H	-6.12582300	4.10357300	2.46879300	C	6.76213300	-1.88200400	-1.37622400
C	-4.87950100	3.21937500	0.94682500	H	7.44310300	-1.42509900	-2.06740000
C	-4.13078900	4.44459800	0.70374900	C	7.14589900	-3.15249800	-0.96785900
C	-4.57989800	5.70645500	1.14885700	C	6.41313400	-3.79975800	-0.00622700
H	-5.51814700	5.78906200	1.67963800	H	6.72756700	-4.76299900	0.36849000
C	-3.87065500	6.85950800	0.88152200	C	5.17518200	-3.26813800	0.37274000
C	-2.68027900	6.80289800	0.13962400	C	4.32827800	-4.09078600	1.22296500
H	-2.14159700	7.71330500	-0.09520600	C	4.86574800	-5.05279000	2.10284300
C	-2.21537600	5.58736800	-0.31109500	H	5.93875900	-5.13190900	2.21752000
H	-1.32695400	5.54452700	-0.92593700	C	4.04847900	-5.86244900	2.86448400
C	-2.91701700	4.39282300	-0.03115800	C	2.65300900	-5.73954200	2.77284800
C	-2.45423200	3.11399800	-0.45572500	H	2.01180200	-6.36207800	3.38566200
C	-3.26989600	1.97399600	-0.41211100	C	2.10021500	-4.80308900	1.92755700
C	-2.45689100	0.80706900	-0.82446600	H	1.02894500	-4.67284200	1.90824900
C	-2.53008000	-0.56925600	-0.68191900	C	2.91959000	-3.96147500	1.14053100
C	-3.52577300	-1.59325900	-0.31107800	C	2.42073900	-2.93489200	0.28926400
C	-4.93717700	-1.50905600	-0.51282300	C	3.26421500	-1.89854100	-0.17083100
C	-4.54923900	2.00900800	0.24341100	C	2.40437800	-0.87740600	-0.78295000
H	4.48603100	-6.57817700	3.55037500	C	2.48533500	0.48453000	-0.93691900
H	8.04351300	-3.59553000	-1.38169500	C	3.47093400	1.49449000	-0.53184700
H	8.40602500	2.31010200	-1.99020500	C	4.90548700	1.43322500	-0.54276700
H	5.30721100	6.62851400	2.20623700	C	4.69420900	-2.02372800	-0.18385700

(*P*,*M*)-**2**$_{\mathbf{meso}}$

C	-1.18985200	-1.37928800	-0.29861900	H	4.83787800	7.69435600	0.49696500
C	0.00000000	-0.75595700	-0.57362700	H	-5.27549800	-7.62353100	0.39571100
C	1.18985200	-1.37928800	-0.29861900	H	8.25553200	-1.98372700	-2.02781300
C	1.23113800	-2.70693600	0.13799800	C	1.20285500	1.25947100	-0.93339900
C	0.00000000	-3.39023100	0.33542700	C	0.00000000	0.58532200	-0.94213300
H	0.00000000	-4.42156600	0.65364000	C	-1.20285500	1.25947100	-0.93339900
C	-1.23113800	-2.70693600	0.13799800	C	-1.23039500	2.65748700	-0.89365400
C	-2.69548600	-2.98167300	0.25373500	C	0.00000000	3.36426500	-0.86524200
C	-3.37479800	-4.23312200	0.35099000	H	0.00000000	4.43098200	-0.69691300
C	-2.73571600	-5.41798000	0.77887800	C	1.23039500	2.65748700	-0.89365400
H	-1.71438100	-5.36760900	1.12772400	C	2.64619800	2.98263700	-0.57995900

C	-3.40354400	-6.62227200	0.79362200	C	3.22107700	4.26135300	-0.32469100
H	-2.90147100	-7.51923100	1.13641900	C	2.61045300	5.46595900	-0.74038700
C	-4.74196400	-6.68058000	0.37515700	H	1.70728100	5.41952100	-1.33266800
C	-5.38941600	-5.53609200	-0.04342600	C	3.17670300	6.68618600	-0.44583700
H	-6.43129500	-5.60296200	-0.32573500	H	2.70525800	7.60162200	-0.78323800
C	-4.73806500	-4.28543200	-0.06713500	C	4.38038200	6.73754200	0.27451300
C	-5.42576800	-3.06828800	-0.47609400	C	5.00056700	5.57257300	0.67766300
C	-6.67252500	-3.11011900	-1.13139900	H	5.94966200	5.64312900	1.19084600
H	-7.10879300	-4.06242200	-1.39946200	C	4.44815200	4.30581700	0.39290300
C	-7.32222300	-1.94754700	-1.47884500	C	5.10514900	3.06621800	0.78473800
C	-6.81656300	-0.71633700	-1.04199700	C	6.16129100	3.08691300	1.71449700
H	-7.40833900	0.18044300	-1.17969800	H	6.43955500	4.01711800	2.18935400
C	-5.60904500	-0.61522800	-0.36157300	C	6.81528600	1.92939600	2.06451000
C	-5.49108800	0.66010300	0.44623400	C	6.50735000	0.74239700	1.39533900
C	-6.50735000	0.74239800	1.39533900	H	7.09937400	-0.14305000	1.58961000
H	-7.09937400	-0.14305000	1.58961000	C	5.49108800	0.66010200	0.44623400
C	-6.81528600	1.92939600	2.06451000	C	5.60904500	-0.61522900	-0.36157300
C	-6.16129000	3.08691400	1.71449800	C	6.81656300	-0.71633700	-1.04199700
H	-6.43955500	4.01711800	2.18935400	H	7.40833900	0.18044300	-1.17969800
C	-5.10514900	3.06621900	0.78473800	C	7.32222200	-1.94754800	-1.47884500
C	-4.44815100	4.30581700	0.39290400	C	6.67252500	-3.11012000	-1.13139900
C	-5.00056600	5.57257300	0.67766400	H	7.10879300	-4.06242200	-1.39946100
H	-5.94966100	5.64312900	1.19084700	C	5.42576800	-3.06828800	-0.47609400
C	-4.38038200	6.73754200	0.27451300	C	4.73806500	-4.28543200	-0.06713500
C	-3.17670300	6.68618600	-0.44583700	C	5.38941600	-5.53609300	-0.04342600
H	-2.70525800	7.60162200	-0.78323700	H	6.43129500	-5.60296300	-0.32573500
C	-2.61045300	5.46595900	-0.74038700	C	4.74196400	-6.68058100	0.37515700
H	-1.70728000	5.41952100	-1.33266800	C	3.40354300	-6.62227300	0.79362200
C	-3.22107700	4.26135300	-0.32469100	H	2.90147000	-7.51923100	1.13641900
C	-2.64619800	2.98263800	-0.57995900	C	2.73571600	-5.41798000	0.77887800
C	-3.37931400	1.79833600	-0.38958100	H	1.71438100	-5.36760900	1.12772400
C	-2.47811500	0.64700900	-0.58823200	C	3.37479800	-4.23312200	0.35099000
C	-2.47338900	-0.71252700	-0.30582200	C	2.69548600	-2.98167300	0.25373500
C	-3.41421600	-1.79541200	0.02708500	C	3.41421600	-1.79541200	0.02708500
C	-4.82075200	-1.79846500	-0.22543100	C	2.47338900	-0.71252700	-0.30582200
C	-4.67135100	1.81285500	0.23579100	C	2.47811500	0.64700900	-0.58823200
H	-8.25553300	-1.98372600	-2.02781300	C	3.37931400	1.79833600	-0.38958100
H	5.27549700	-7.62353100	0.39571200	C	4.67135200	1.81285500	0.23579100
H	7.59657400	1.94172000	2.81490200	C	4.82075200	-1.79846500	-0.22543100
H	-7.59657300	1.94172000	2.81490300	H	-4.83787700	7.69435600	0.49696500

TS$_2$

C	-1.08400000	-1.47209300	-0.83771000	H	4.24935100	7.81480500	1.22546000
C	0.07265300	-0.77006500	-1.12889700	H	-4.48602700	-6.57817600	3.55037700
C	1.29714900	-1.31680900	-0.80376800	H	8.41153500	-1.42681900	-2.23777700

C	1.40883800	-2.62082300	-0.29168600	C	1.15163200	1.36426500	-1.14451100
C	0.23011700	-3.31272200	0.05137200	C	-0.00551500	0.61915900	-1.31463600
H	0.28599000	-4.27591700	0.53314100	C	-1.24778200	1.21543000	-1.15319600
C	-1.03127500	-2.69596600	-0.16249400	C	-1.35662700	2.54475700	-0.72536000
C	-2.42073800	-2.93489300	0.28926400	C	-0.18148100	3.32530500	-0.61356700
C	-2.91958900	-3.96147500	1.14053100	H	-0.22960300	4.33919600	-0.25041400
C	-2.10021300	-4.80308900	1.92755800	C	1.07803700	2.73155300	-0.83860800
H	-1.02894300	-4.67284000	1.90824900	C	2.45423100	3.11399800	-0.45572600
C	-2.65300600	-5.73954100	2.77284900	C	2.91701600	4.39282300	-0.03115900
H	-2.01179900	-6.36207700	3.38566400	C	2.21537500	5.58736800	-0.31109700
C	-4.04847600	-5.86244900	2.86448600	H	1.32695300	5.54452700	-0.92594000
C	-4.86574600	-5.05279000	2.10284500	C	2.68027800	6.80289800	0.13962200
H	-5.93875700	-5.13190900	2.21752200	H	2.14159600	7.71330500	-0.09520800
C	-4.32827600	-4.09078600	1.22296600	C	3.87065400	6.85950800	0.88152100
C	-5.17518100	-3.26813900	0.37274100	C	4.57989600	5.70645600	1.14885700
C	-6.41313300	-3.79975900	-0.00622500	H	5.51814400	5.78906300	1.67963800
H	-6.72756500	-4.76300000	0.36849300	C	4.13078700	4.44459900	0.70374900
C	-7.14589800	-3.15250000	-0.96785700	C	4.87949800	3.21937500	0.94682600
C	-6.76213300	-1.88200600	-1.37622200	C	5.92944900	3.21700900	1.88293300
H	-7.44310300	-1.42510100	-2.06739900	H	6.12581700	4.10357200	2.46879700
C	-5.63881800	-1.17780400	-0.91135100	C	6.68303600	2.08800200	2.09712500
C	-5.73159100	0.34803800	-1.06988200	C	6.47171800	0.96821200	1.29174100
C	-6.92557600	0.80207300	-1.65489800	H	7.12878100	0.11390600	1.39216400
H	-7.53590800	0.13580900	-2.23271000	C	5.46177400	0.91252700	0.33209500
C	-7.46687000	2.07211100	-1.50584800	C	5.66194800	-0.28118700	-0.57334500
C	-6.83189000	2.98344200	-0.70148100	C	6.88540800	-0.28256000	-1.23088700
H	-7.26674900	3.95616500	-0.52309800	H	7.43139300	0.64971400	-1.31029500
C	-5.54245400	2.69451400	-0.24011900	C	7.46775700	-1.46513200	-1.70715700
C	-4.81259900	3.76849900	0.41615900	C	6.88266100	-2.67615100	-1.41353200
C	-5.47634500	4.81038200	1.09597500	H	7.38122400	-3.59082500	-1.70322100
H	-6.55176800	4.77399700	1.20955600	C	5.62096800	-2.73166000	-0.78682700
C	-4.77601200	5.85191000	1.66875000	C	4.99463300	-3.99816400	-0.43479400
C	-3.37575100	5.89042300	1.57945300	C	5.71615800	-5.20984400	-0.43920700
H	-2.82375800	6.69865200	2.04450900	H	6.76735300	-5.20588100	-0.69360900
C	-2.70035000	4.88212100	0.92798100	C	5.12372100	-6.40479800	-0.08593300
H	-1.62160600	4.89203500	0.91753800	C	3.77028200	-6.43915400	0.28359800
C	-3.39788200	3.80266300	0.33826600	H	3.30646800	-7.37780300	0.56271600
C	-2.76741700	2.69678900	-0.30159600	C	3.03549600	-5.27441200	0.29682100
C	-3.47093400	1.49448900	-0.53184800	H	1.99659400	-5.30422200	0.59046700
C	-2.48533500	0.48452900	-0.93691900	C	3.62033800	-4.03661000	-0.05123000
C	-2.40437800	-0.87740600	-0.78295100	C	2.87662500	-2.81944100	-0.12616100
C	-3.26421500	-1.89854200	-0.17083100	C	3.52577400	-1.59325800	-0.31107800
C	-4.69420900	-2.02372900	-0.18385700	C	2.53008100	-0.56925600	-0.68192000
C	-4.90548800	1.43322400	-0.54276700	C	2.45689100	0.80706900	-0.82446700

H	-8.04351300	-3.59553200	-1.38169300	C	3.26989600	1.97399700	-0.41211200
H	5.70930100	-7.31656100	-0.08484900	C	4.54923700	2.00900900	0.24341200
H	7.46179600	2.07656500	2.85012200	C	4.93717700	-1.50905400	-0.51282300
H	-8.40602700	2.31010000	-1.99020400	H	-5.30721100	6.62851400	2.20623700
				(*M*,*M*)-**2**$_{\mathbf{rac}}$			
C	1.04868200	1.48234400	-0.70996200	H	-3.99188900	-7.91114600	1.86923100
C	-0.07096300	0.69245800	-0.85311800	H	3.99188900	7.91114600	1.86923400
C	-1.32929800	1.21668900	-0.67582100	H	-8.42920600	0.74319400	-2.26338300
C	-1.52136200	2.58163900	-0.43365800	C	-1.04868200	-1.48234400	-0.70996300
C	-0.37644100	3.40467300	-0.29121100	C	0.07096300	-0.69245700	-0.85311800
H	-0.48403900	4.43876100	-0.00168500	C	1.32929800	-1.21668900	-0.67582100
C	0.92320200	2.84232800	-0.41147400	C	1.52136200	-2.58163900	-0.43365800
C	2.30025300	3.25723600	-0.04085200	C	0.37644100	-3.40467300	-0.29121200
C	2.73863400	4.52504800	0.44062100	H	0.48403900	-4.43876100	-0.00168600
C	2.00182900	5.71328500	0.23558200	C	-0.92320200	-2.84232800	-0.41147500
H	1.10605200	5.67865800	-0.36860800	C	-2.30025300	-3.25723600	-0.04085400
C	2.43907700	6.91547400	0.74564900	C	-2.73863400	-4.52504800	0.44062000
H	1.87230400	7.82164900	0.56735400	C	-2.00182900	-5.71328500	0.23558000
C	3.63581600	6.96557800	1.47759200	H	-1.10605200	-5.67865800	-0.36861000
C	4.37833000	5.81954100	1.67627100	C	-2.43907700	-6.91547400	0.74564700
H	5.31929200	5.89776900	2.20318200	H	-1.87230400	-7.82165000	0.56735200
C	3.95998600	4.57191200	1.16692800	C	-3.63581600	-6.96557900	1.47759000
C	4.74776400	3.35922200	1.33896900	C	-4.37833000	-5.81954200	1.67626800
C	5.80011600	3.32532500	2.27236800	H	-5.31929200	-5.89777000	2.20318000
H	5.97739700	4.17891400	2.91126900	C	-3.95998700	-4.57191200	1.16692600
C	6.57806800	2.20115700	2.41724900	C	-4.74776400	-3.35922300	1.33896700
C	6.39769200	1.12974100	1.53940300	C	-5.80011600	-3.32532600	2.27236600
H	7.08317600	0.29267200	1.57903600	H	-5.97739800	-4.17891500	2.91126700
C	5.39126400	1.11172600	0.57615600	C	-6.57806800	-2.20115800	2.41724700
C	5.64348900	0.02325100	-0.44486400	C	-6.39769200	-1.12974100	1.53940100
C	6.85697600	0.17201500	-1.10579000	H	-7.08317600	-0.29267300	1.57903400
H	7.34819300	1.13684200	-1.07189700	C	-5.39126400	-1.11172600	0.57615500
C	7.49603500	-0.90302300	-1.73672200	C	-5.64348900	-0.02325100	-0.44486500
C	6.97749100	-2.17110600	-1.60595700	C	-6.85697600	-0.17201400	-1.10579200
H	7.51703800	-3.01082000	-2.02158700	H	-7.34819300	-1.13684200	-1.07189900
C	5.73050100	-2.37689100	-0.98250400	C	-7.49603400	0.90302400	-1.73672300
C	5.18010200	-3.71336600	-0.80431000	C	-6.97749100	2.17110700	-1.60595800
C	5.96763400	-4.87061900	-0.97664700	H	-7.51703800	3.01082000	-2.02158800
H	7.01353400	-4.77336500	-1.23406400	C	-5.73050100	2.37689100	-0.98250500
C	5.44782100	-6.13393700	-0.78267200	C	-5.18010100	3.71336600	-0.80431000
C	4.10511400	-6.29488800	-0.40715900	C	-5.96763400	4.87061900	-0.97664700
H	3.70082400	-7.28724100	-0.24612700	H	-7.01353400	4.77336500	-1.23406400
C	3.30688200	-5.18630900	-0.23321600	C	-5.44782000	6.13393700	-0.78267200
H	2.27942900	-5.31192800	0.07518400	C	-4.10511400	6.29488800	-0.40715900

C	3.81588700	-3.88207400	-0.42218100	H	-3.70082400	7.28724100	-0.24612600
C	3.00414100	-2.71208700	-0.32125900	C	-3.30688200	5.18630900	-0.23321600
C	3.58539700	-1.43437000	-0.32795100	H	-2.27942900	5.31192800	0.07518500
C	2.52977700	-0.42041500	-0.50017900	C	-3.81588700	3.88207400	-0.42218100
C	2.38818700	0.96019000	-0.49455100	C	-3.00414100	2.71208700	-0.32125900
C	3.16001000	2.14439800	-0.06531900	C	-3.58539700	1.43437000	-0.32795200
C	4.44821200	2.18634200	0.56681100	C	-2.52977700	0.42041500	-0.50017900
C	4.98648200	-1.24175200	-0.53606200	C	-2.38818700	-0.96019000	-0.49455200
H	7.35688200	2.16107200	3.16922100	C	-3.16001000	-2.14439800	-0.06532100
H	-6.08349300	7.00253900	-0.90795800	C	-4.44821300	-2.18634200	0.56681000
H	-7.35688200	-2.16107300	3.16921900	C	-4.98648200	1.24175300	-0.53606200
H	8.42920600	-0.74319400	-2.26338100	H	6.08349400	-7.00253900	-0.90795800

Table S5. TD-DFT singlet excitation energies E, excitation wavelengths λ, oscillator strengths *f*, and orbital contributions of $\mathbf{2_{meso}}$ at the B3LYP/6-311G(d,p) level in $CHCl_3$ solution.

Excited state	Energy (eV)	Wavelength (nm)	*f*	Contributions
S_1	1.3266	934.60	0.00000	HOMO→LUMO: 99.4%
S_5	2.1640	572.94	0.40150	HOMO-4→LUMO: 95.8%
S_7	2.4517	505.71	0.16600	HOMO-6→LUMO: 80.1%<br>HOMO→LUMO+1: 11.2%
S_8	2.5256	490.91	0.12210	HOMO→LUMO+1: 83.1%<br>HOMO-6→LUMO: 13.2%
S_{10}	2.6264	472.07	0.10060	HOMO-1→LUMO+1: 51.5%<br>HOMO-8→LUMO: 46.4%
S_{13}	2.8218	439.38	0.84240	HOMO-8→LUMO: 49.4%<br>HOMO-1→LUMO+1: 42.4%
S_{17}	3.1517	393.39	1.14030	HOMO-2→LUMO+2: 84.1%<br>HOMO-9→LUMO: 6.0%
S_{23}	3.3504	370.06	0.27060	HOMO→LUMO+3: 45.5%<br>HOMO-4→LUMO+2: 13.3%<br>HOMO-13→LUMO: 12.9%<br>HOMO-2→LUMO+5: 7.1%
S_{24}	3.3666	368.28	0.16840	HOMO→LUMO+4: 31.0%<br>HOMO-1→LUMO+3: 30.4%<br>HOMO-5→LUMO+1: 18.7%<br>HOMO-2→LUMO+6: 5.4%
S_{31}	3.5310	351.13	0.46330	HOMO-4→LUMO+2: 63.4%<br>HOMO→LUMO+3: 23.3%<br>HOMO→LUMO+5: 6.3%

Table S6. TD-DFT singlet excitation energies E, excitation wavelengths λ, oscillator strengths *f*, and orbital contributions of $\mathbf{2_{rac}}$ at the B3LYP/6-311G(d,p) level in $CHCl_3$ solution.

Excited state	Energy (eV)	Wavelength (nm)	*f*	Contributions
S_1	1.3446	922.09	0.00000	HOMO→LUMO: 99.2%
S_5	2.1851	567.41	0.38620	HOMO-4→LUMO: 94.5%
S_7	2.4728	501.39	0.15550	HOMO-6→LUMO: 77.7%<br>HOMO→LUMO+1: 12.9%
S_8	2.5368	488.74	0.12480	HOMO→LUMO+1: 80.9%<br>HOMO-6→LUMO: 15.1%
S_{14}	2.8427	436.15	0.86640	HOMO-8→LUMO: 47.3%<br>HOMO-1→LUMO+1: 43.7%
S_{17}	3.1489	393.74	1.18150	HOMO-2→LUMO+2: 86.5%<br>HOMO-9→LUMO: 6.5%
S_{21}	3.3459	370.56	0.34980	HOMO→LUMO+3: 47.7%<br>HOMO-4→LUMO+2: 11.3%<br>HOMO→LUMO+4: 8.5%<br>HOMO-2→LUMO+5: 6.7%<br>HOMO-3→LUMO+2: 6.0%
S_{24}	3.3714	367.75	0.17000	HOMO→LUMO+4: 33.0%<br>HOMO-1→LUMO+3: 23.2%<br>HOMO-5→LUMO+1: 17.8%<br>HOMO-2→LUMO+6: 5.6%<br>HOMO-1→LUMO+4: 5.1%
S_{30}	3.5174	352.49	0.20710	HOMO-4→LUMO+2: 45.6%<br>HOMO-1→LUMO+4: 18.6%<br>HOMO-3→LUMO+2: 15.6%<br>HOMO→LUMO+3: 9.1%
S_{31}	3.5427	349.97	0.25480	HOMO-4→LUMO+2: 26.6%<br>HOMO-1→LUMO+4: 25.3%<br>HOMO→LUMO+4: 23.0%<br>HOMO-3→LUMO+2: 6.1%

5. Chiral HPLC Analysis and Thermal Isomerization of 2_{meso} and 2_{rac}

HPLC analysis was conducted on a Shimadzu LC-20A instrument equipped with equipped with a CHIRALPAK® IF column (eluent: n-hexane/dichloromethane = 93:7, 2.0 mL·min^{-1}, 25 °C, Detector: PDA Multi 525nm 4nm).

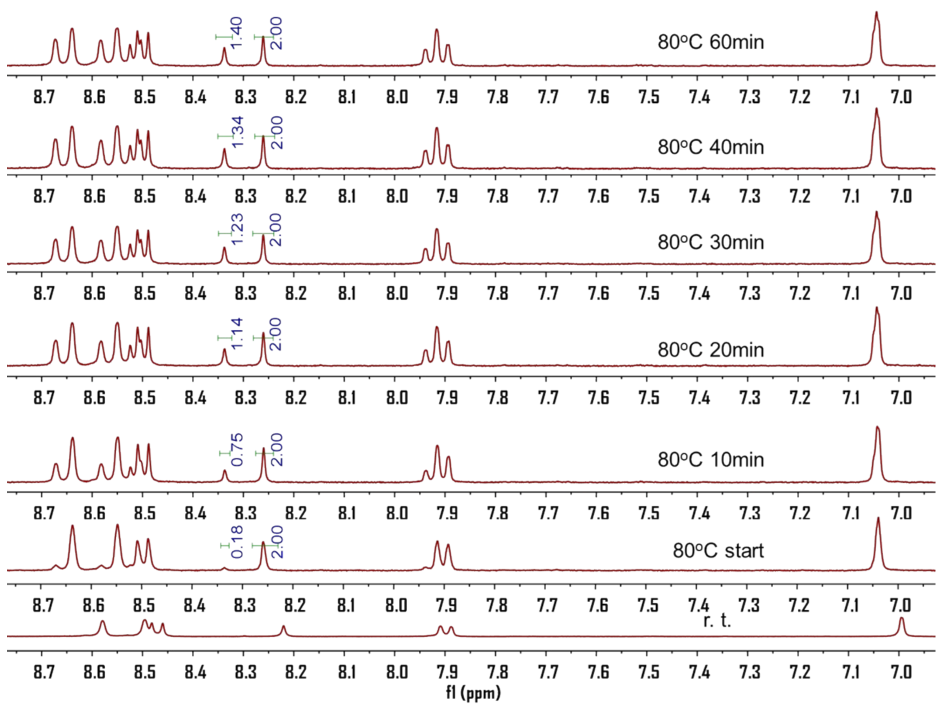

Fig. S6 Selected ¹H NMR spectra of during the progress of thermal $\mathbf{2_{meso}}$ to $\mathbf{2_{rac}}$ isomerization in 1,1,2,2-tetrachlorothane-d_2 at 80 °C.

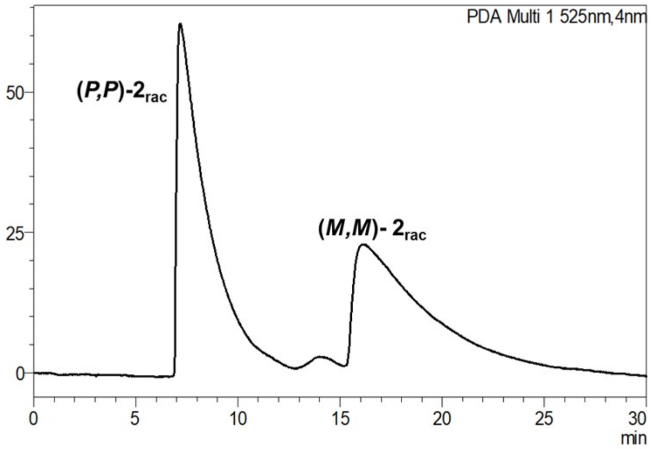

Fig. S7 The HPLC analysis spectra of $\mathbf{2_{rac}}$.

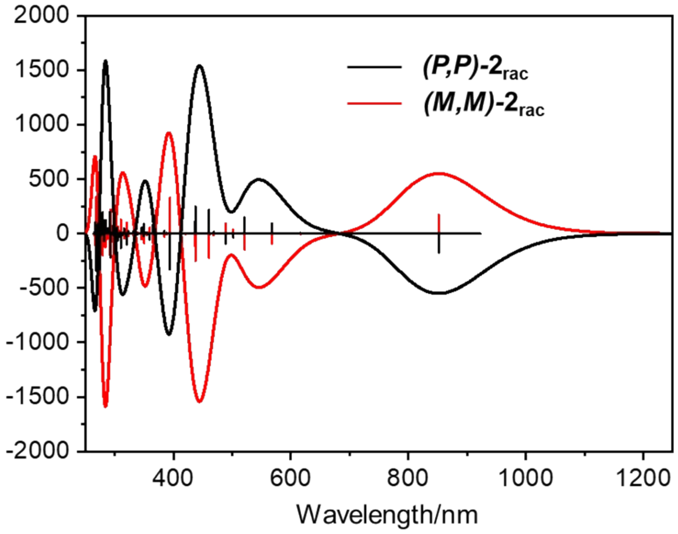

Fig. S8 Calculated CD spectra of *(P,P)*-$\mathbf{2_{rac}}$ and *(M,M)*-$\mathbf{2_{rac}}$ at the B3LYP/6-311G(d,p) level in $CHCl_3$ solution.

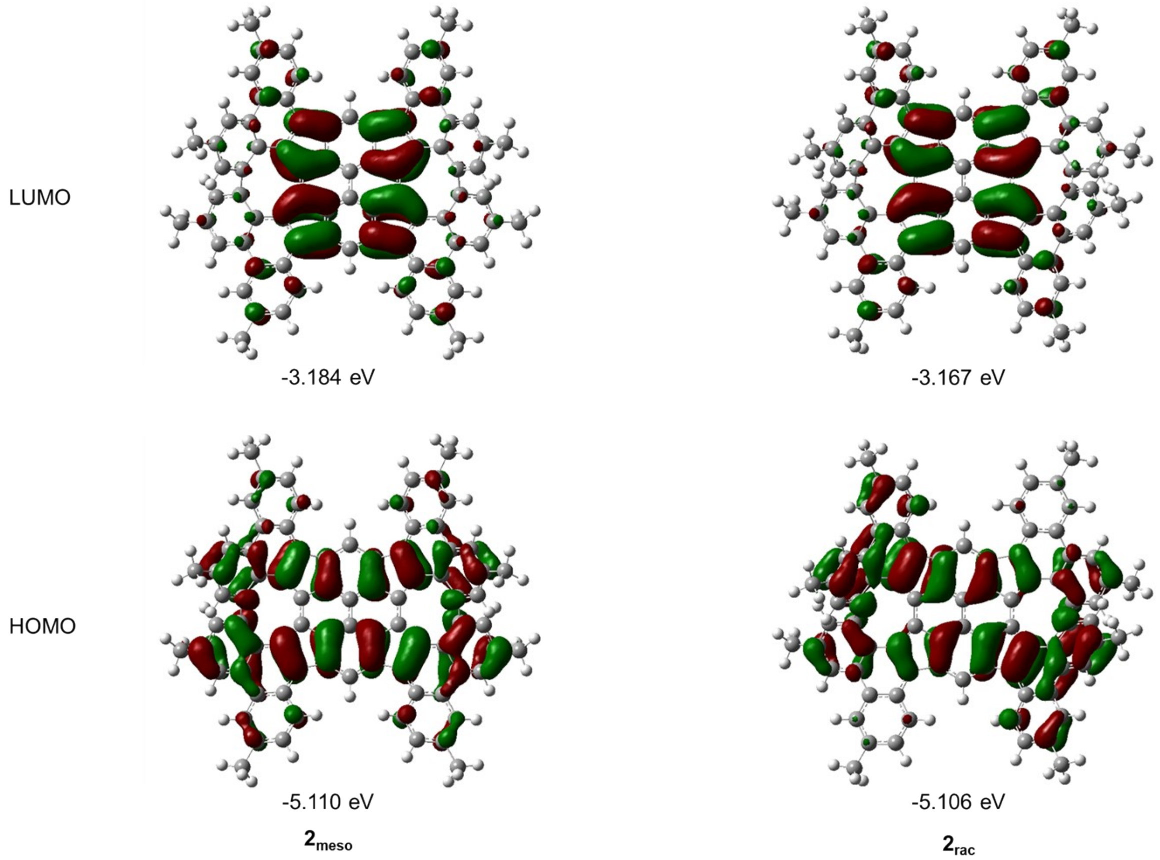

Fig. S9 Frontier orbitals and frontier orbital energies of $\mathbf{2_{meso}}$ and $\mathbf{2_{rac}}$ at the optimized S_0 geometry at the B3LYP/6-311G(d,p) level.

6. Binding Behaviors

The association behaviors of $\mathbf{2_{meso}}$ and guest were analyzed by NMR titrations. A deuterium solution of $\mathbf{2_{meso}}$ (4.0×10^{-4} M) and a solution of guest (4.0×10^{-4} M) were prepared. The solutions were mixed with different ratios.

A solution of $\mathbf{2_{meso}}$ at a concentration of approximately. Those solutions were utilized as solvents in the guest solution, to ensure working at constant concentration of host. Nonlinear curve regression of $\mathbf{2_{meso}}$ upon addition of guest by using 1:1 binding model associated with the program BindFit[10].

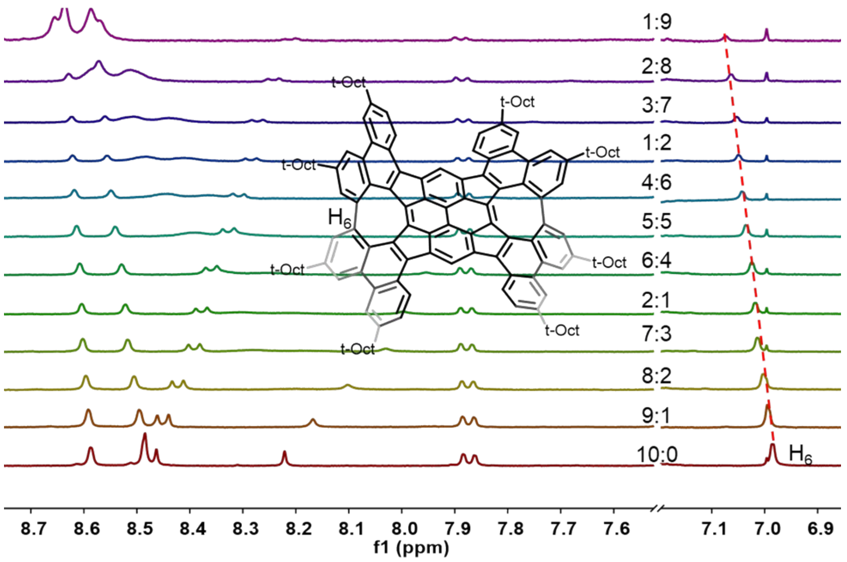

Fig. S10 Part of the ^{1}H NMR spectra of $\mathbf{2_{meso}}$ and PDI mixed in different ratio in Chloroform-*d* at 298 K (total concentration of 4.0×10^{-4} M).

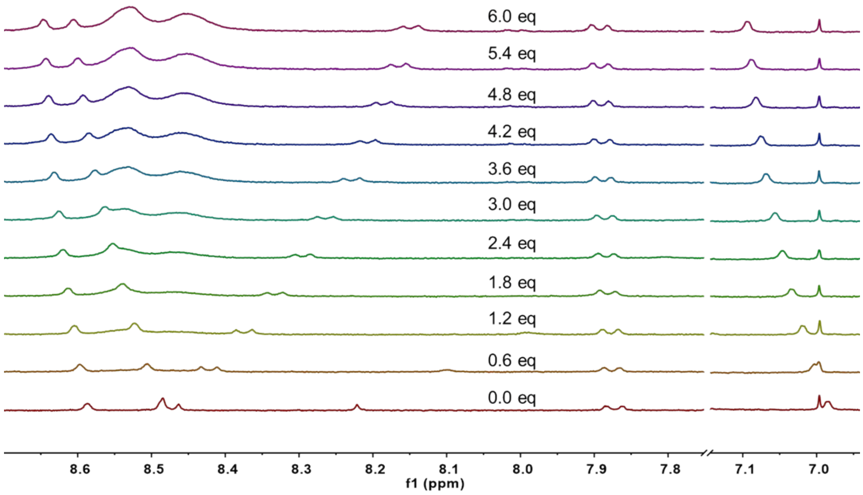

Fig. S11 Part of the ^{1}H NMR spectra of $\mathbf{2}_{meso}$ titration with PDI. The concentration [$\mathbf{2}_{meso}$] was kept to be 1.0×10^{-4} M for all entries and the concentration [PDI] was changed from 0.0 to 6.0×10^{-4} M. (400MHz, Chloroform-*d*, 298 K).

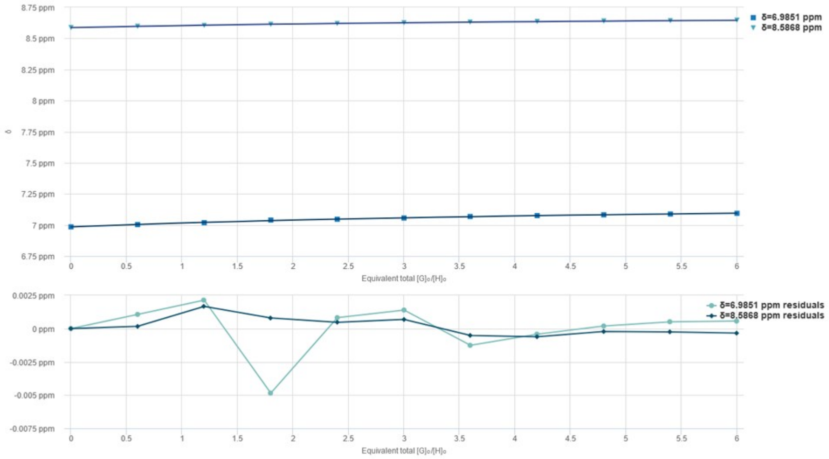

Fig. S12 Nonlinear curve regression of protons of $\mathbf{2}_{meso}$ upon addition of PDI by using 1:1 binding model associated with the program BindFit[10].

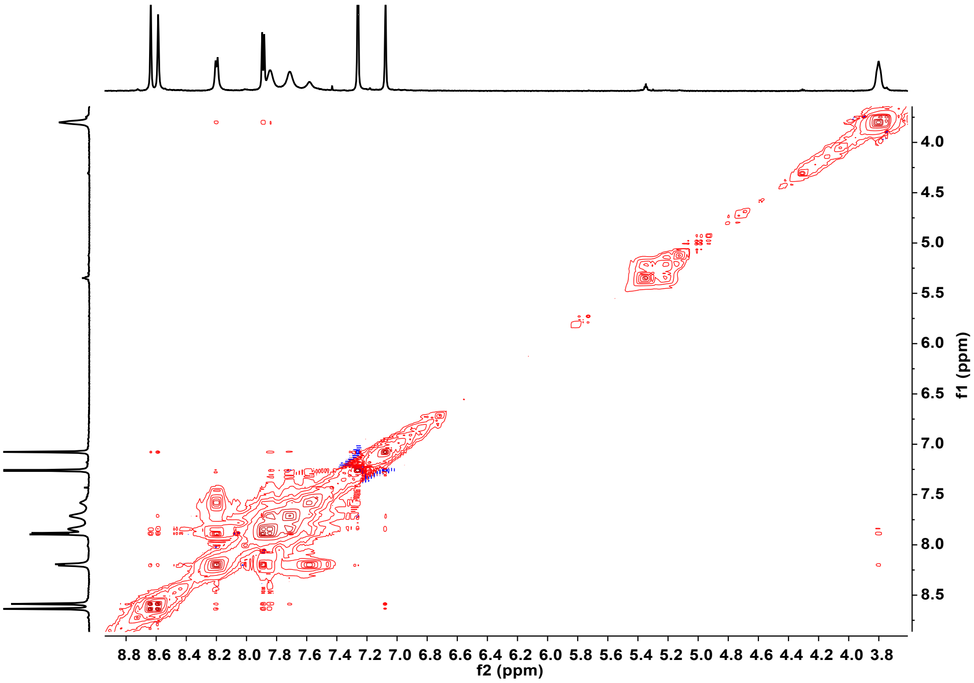

Fig. S13 2D ^{1}H–^{1}H NOESY NMR spectrum of $\mathbf{2}_{meso}$ and PDI (600 MHz, Chloroform-*d*, 298 K).

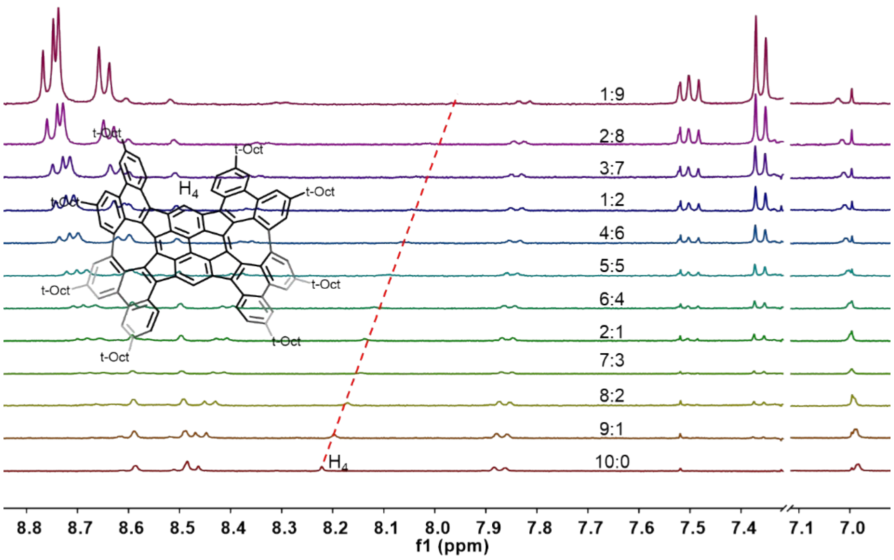

Fig. S14 Part of the ^{1}H NMR spectra of $\mathbf{2_{meso}}$ and TDI mixed in different ratio in Chloroform-*d* at 298 K (total concentration of 4.0×10^{-4} M).

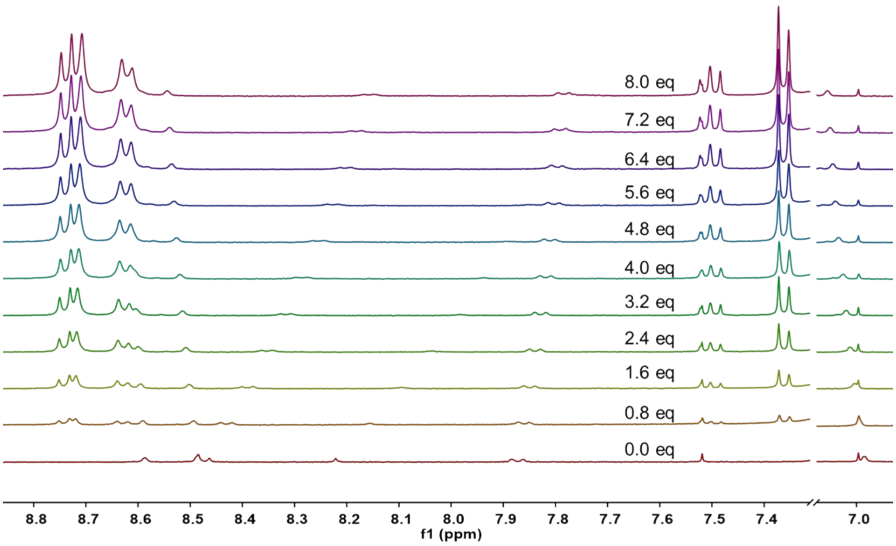

Fig. S15 Part of the ^{1}H NMR spectra of $\mathbf{2_{meso}}$ titration with TDI. The concentration [$\mathbf{2_{meso}}$] was kept to be 1.0×10^{-4} M for all entries and the concentration [TDI] was changed from 0.0 to 8.0×10^{-4} M. (400MHz, Chloroform-*d*, 298 K).

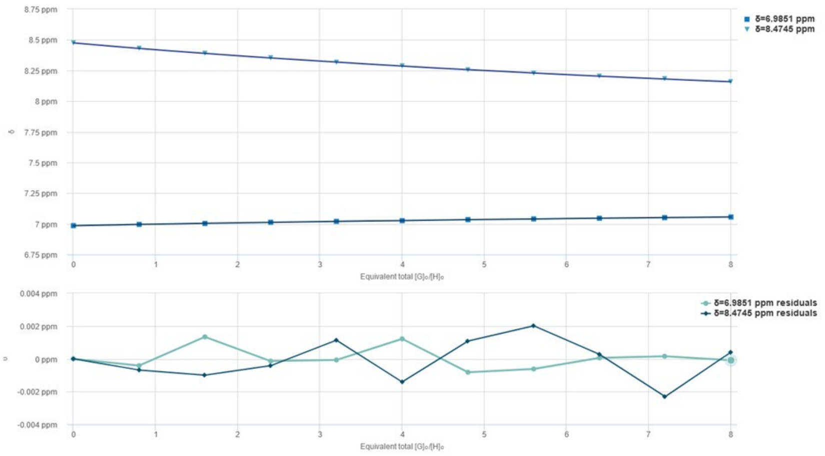

Fig. S16 Nonlinear curve regression of $\mathbf{2_{meso}}$ upon addition of TDI by using 1:1 binding model associated with the program BindFit[10].

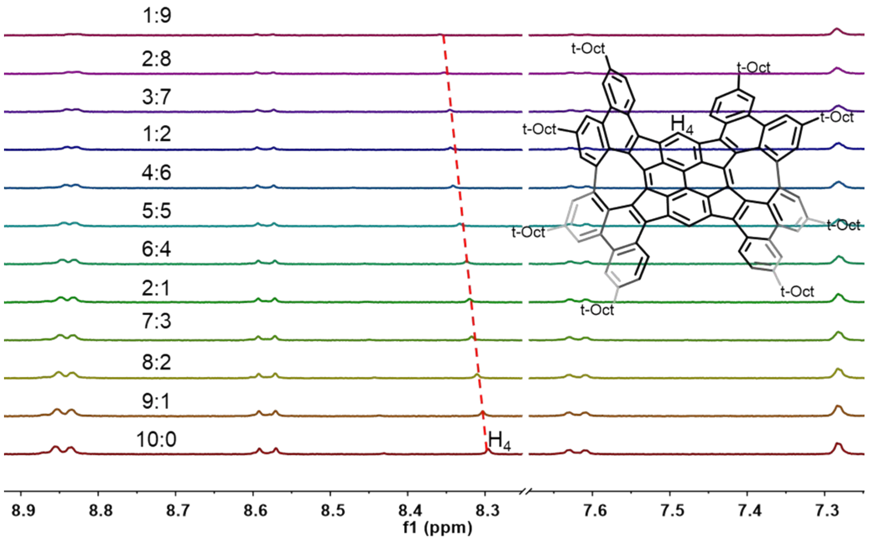

Fig. S17 Part of the ^{1}H NMR spectra of $\mathbf{2_{meso}}$ and C_{60} mixed in different ratio in toluene-d_8 at 298 K (total concentration of 4.0×10^{-4} M).

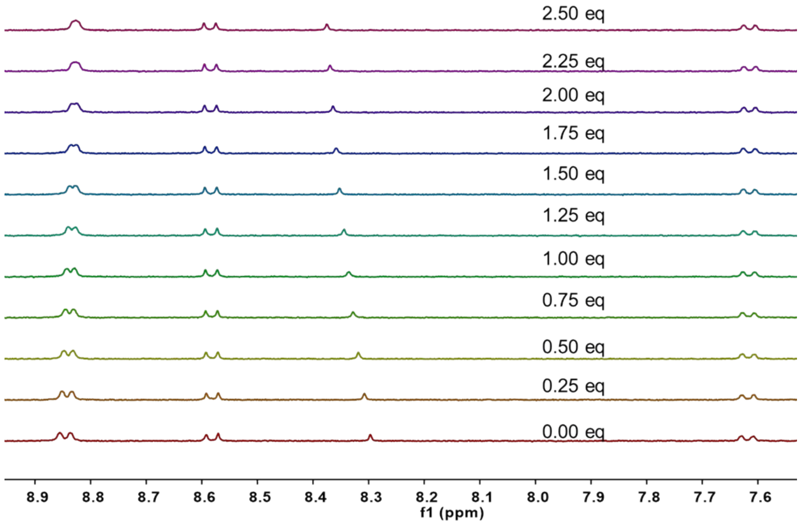

Fig. S18 Part of the ^{1}H NMR spectra of $\mathbf{2_{meso}}$ titration with C_{60}. The concentration [$\mathbf{2_{meso}}$] was kept to be 2.0×10^{-4} M for all entries and the concentration [C_{60}] was changed from 0.0 to 5.0×10^{-4} M. (400MHz, toluene-d_8, 298 K).

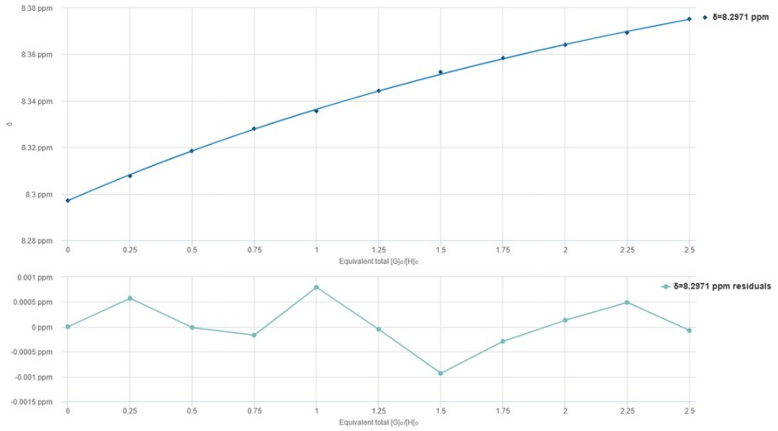

Fig. S19 Nonlinear curve regression of $\mathbf{2_{meso}}$ upon addition of C_{60} by using 1:1 binding model associated with the program BindFit[10].

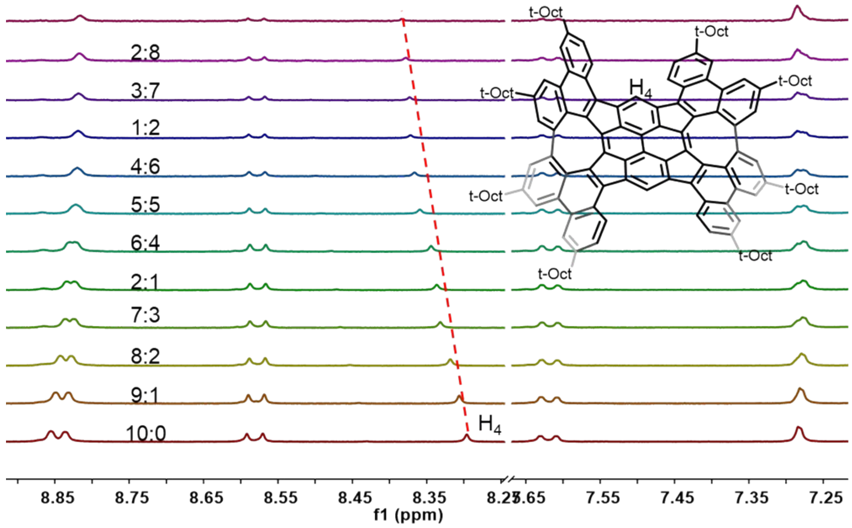

Fig. S20 Part of the ^{1}H NMR spectra of $\mathbf{2_{meso}}$ and C_{70} mixed in different ratio in toluene-d_8 at 298 K (total concentration of 4.0×10^{-4} M).

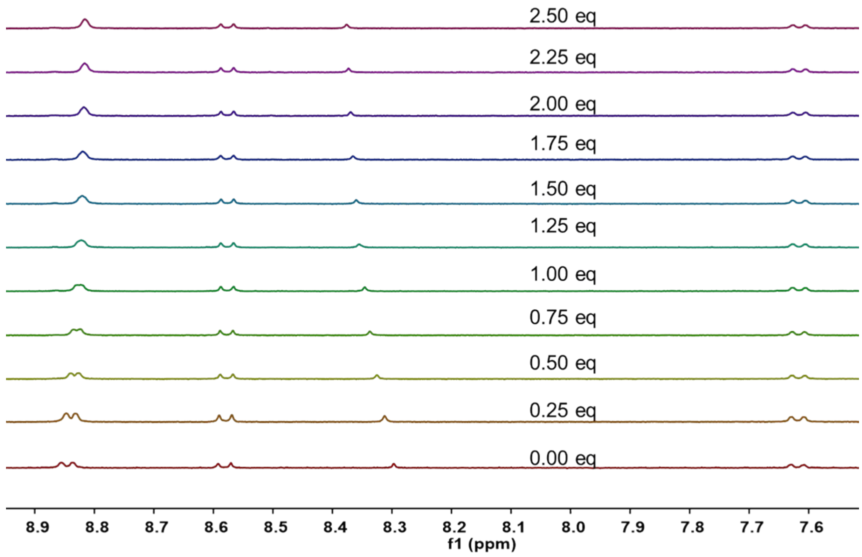

Fig. S21 Part of the ^{1}H NMR spectra of $\mathbf{2_{meso}}$ titration with C_{70}. The concentration [$\mathbf{2_{meso}}$] was kept to be 2.0×10^{-4} M for all entries and the concentration [C_{70}] was changed from 0.0 to 5.0×10^{-4} M. (400MHz, toluene-d_8, 298 K).

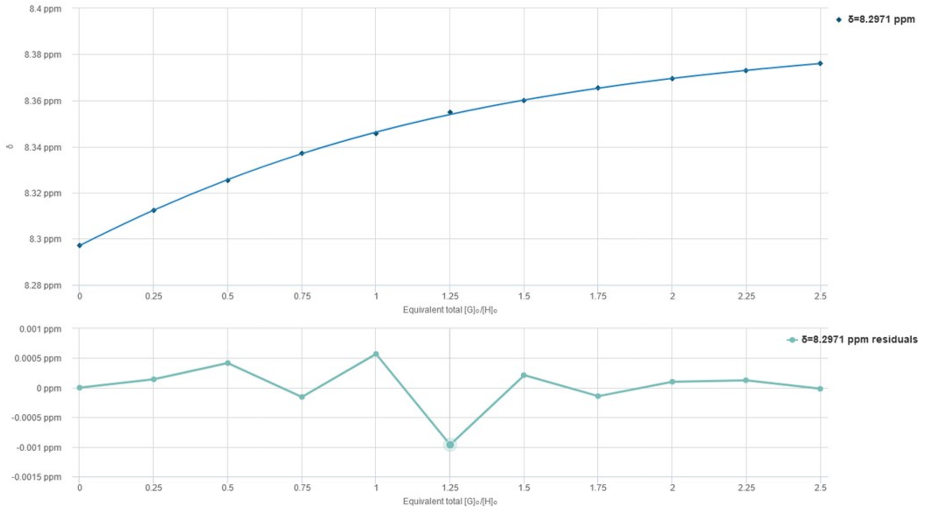

Fig. S22 Nonlinear curve regression of $\mathbf{2_{meso}}$ upon addition of C_{70} by using 1:1 binding model associated with the program BindFit[10].

7. X-ray Crystallographic Data for Compounds

Single crystals of **1** suitable for X-ray diffraction analysis were grown by slow diffusion of methanol into chloroform solution at room temperature, $\mathbf{2_{meso}}$ was grown by slow diffusion of chlorobenzene into methanol solution at room temperature, $\mathbf{2_{rac}}$ was grown by slow diffusion of toluene into methanol solution at room temperature. The X-ray diffraction data were collected on a MM007HF Saturn724+ diffractometer with graphite monochromated Cu Kα (1.54184 Å) and Mo Kα (0.71073 Å). These structures were refined using the SHELXL-2018. Crystallographic data have been deposited with the Cambridge Crystallographic Data Centre as supplementary publication No. CCDC 2353253 (**1**), 2353606 ($\mathbf{2_{meso}}$) and 2353623 ($\mathbf{2_{rac}}$). The single crystal X-ray crystallographic data were summarized in Table S7. (**NOTE**: The large Rint value in the checkcif report of $\mathbf{2_{rac}}$ was due to the weak reflection intensity at higher angles; Due to the weak diffraction of $\mathbf{2_{rac}}$ crystals, some reflections with large estimated standard deviations were omitted during refinement.)

Table S7. Crystal data and structure refinement for **1**, $\mathbf{2_{meso}}$ and $\mathbf{2_{rac}}$.

Identification code	**1**	$\mathbf{2_{meso}}$	$\mathbf{2_{rac}}$
Empirical formula	$C_{136}H_{170}$ $CHCl_3$	$C_{142}H_{163}Cl$	$C_{136}H_{158}$
Formula weight	1924.08	1905.16	1792.61
Crystal system	Monoclinic	Monoclinic	orthorhombic
Space group	*C2/c*	*P 2₁/c*	*Fd₃*
Radiation type	Cu Kα	Cu Kα	Mo Kα
Radiation wavelength(Å)	1.54178	1.54184	0.71073
T(K)	169.99(10)	169.99(10)	117.40(14)
a(Å)	38.6708(6)	23.7294(2)	46.521(2)
b(Å)	24.1800(2)	16.10060(10)	45.590(2)
c(Å)	26.6822(6)	31.9392(2)	24.322(2)
α(deg)	90	90	90
β(deg)	108.601(2)	101.5800(10)	90
γ(deg)	90	90	90
V(Å3)	23646.2(7)	11954.24(15)	51585(6)
Z	8	4	16
ρcalc (g cm^{-3})	1.081	1.059	0.923
θ, range(°)	7.116 – 135.236	6.948 -133.580	6.022 - 50.196
R(int)	9.41%	13.40%	19.1%
μ(mm^{-1})	1.054	0.640	0.052
F(000)	8352.0	4128.0	15584.0
Crystal size (mm^3)	0.28 × 0.13 × 0.05	0.36 × 0.28 × 0.021	0.34 × 0.33 × 0.12
Index ranges	-45 ≤ h ≤ 45, -28 ≤ k ≤ 28, -30 ≤ l ≤ 31	-25 ≤ h ≤ 27, -18 ≤ k ≤ 19, -38 ≤ l ≤ 37	-41 ≤ h ≤ 55, -52 ≤ k ≤ 54, -29 ≤ l ≤ 27
Absorption correction	spherical harmonics	spherical harmonics	spherical harmonics
Max. transmission	1.000	1.000	1.000
Min. transmission	0.802	0.475	0.967
Goodness-of-fit on F^2	2.231	1.035	1.244
Final R indices [I>2sigma(I)]	R1 = 0.1161, wR2 = 0.0.3203	R1 = 0.0701, wR2 = 0.2011	R1 = 0.1200, wR2 = 0.2732
R indices (all data)	R1 = 0.1397, wR2 = 0.3350	R1 = 0.0900, wR2 = 0.2191	R1 = 0.2174, wR2 = 0.3108
Largest diff. peak and hole (e.$Å^{-3}$)	0.99 and -0.35	0.52 and -0.64	0.45/-0.24

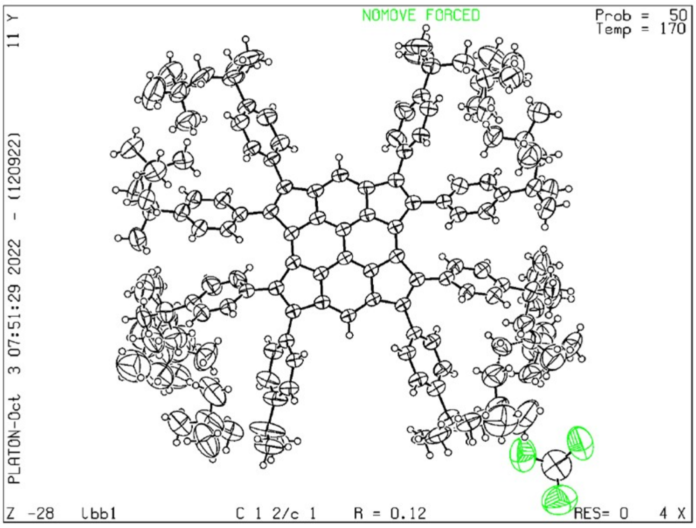

Fig. S23 Thermal ellipsoids drawn at the 50% probability level of **1**. Chlorine atoms are depicted by green ellipsoids; Hydrogens are depicted by white circles.

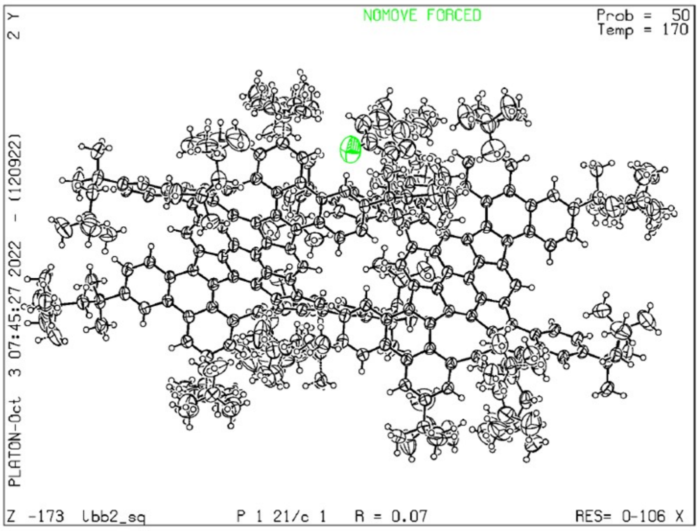

Fig. S24 Thermal ellipsoids drawn at the 50% probability level of $\mathbf{2}_{\mathbf{meso}}$. Chlorine atoms are depicted by green ellipsoids; Hydrogens are depicted by white circles.

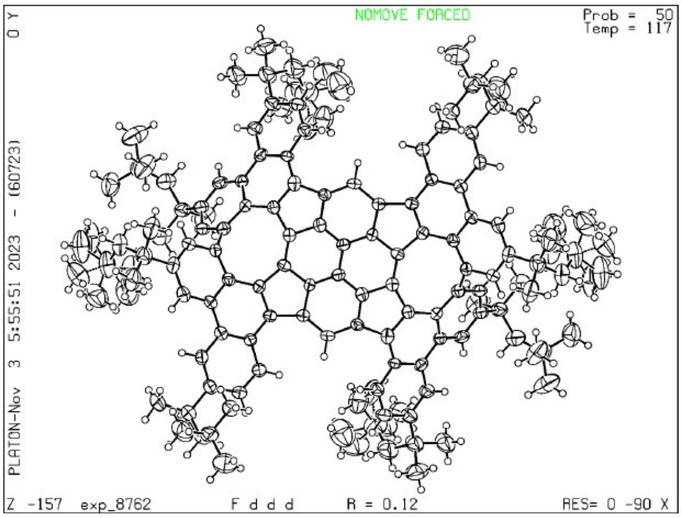

Fig. S25 Thermal ellipsoids drawn at the 50% probability level of $\mathbf{2}_{\mathbf{rac}}$. Hydrogens are depicted by white circles.

8. ¹H NMR, ¹³C NMR, and HRMS Spectra of Compounds

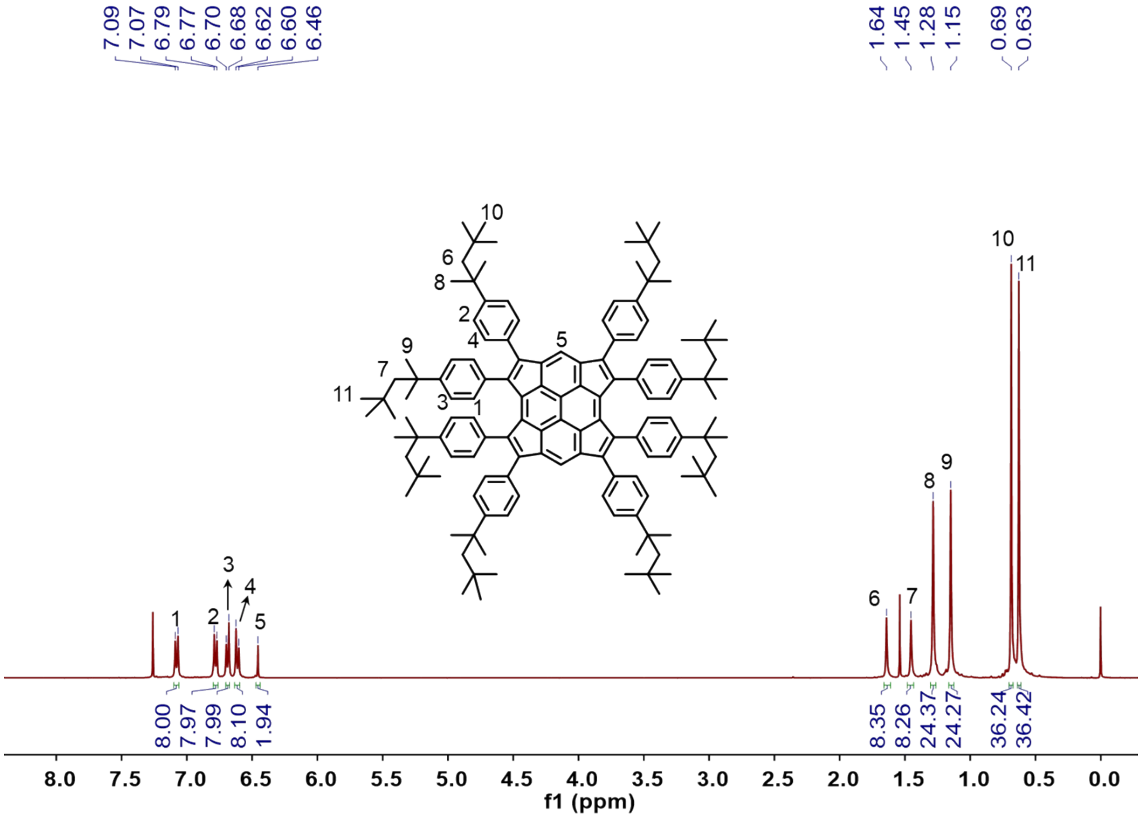

Fig. S26 ¹H NMR spectrum of **1** (Chloroform-*d*, 400 MHz, 298 K)

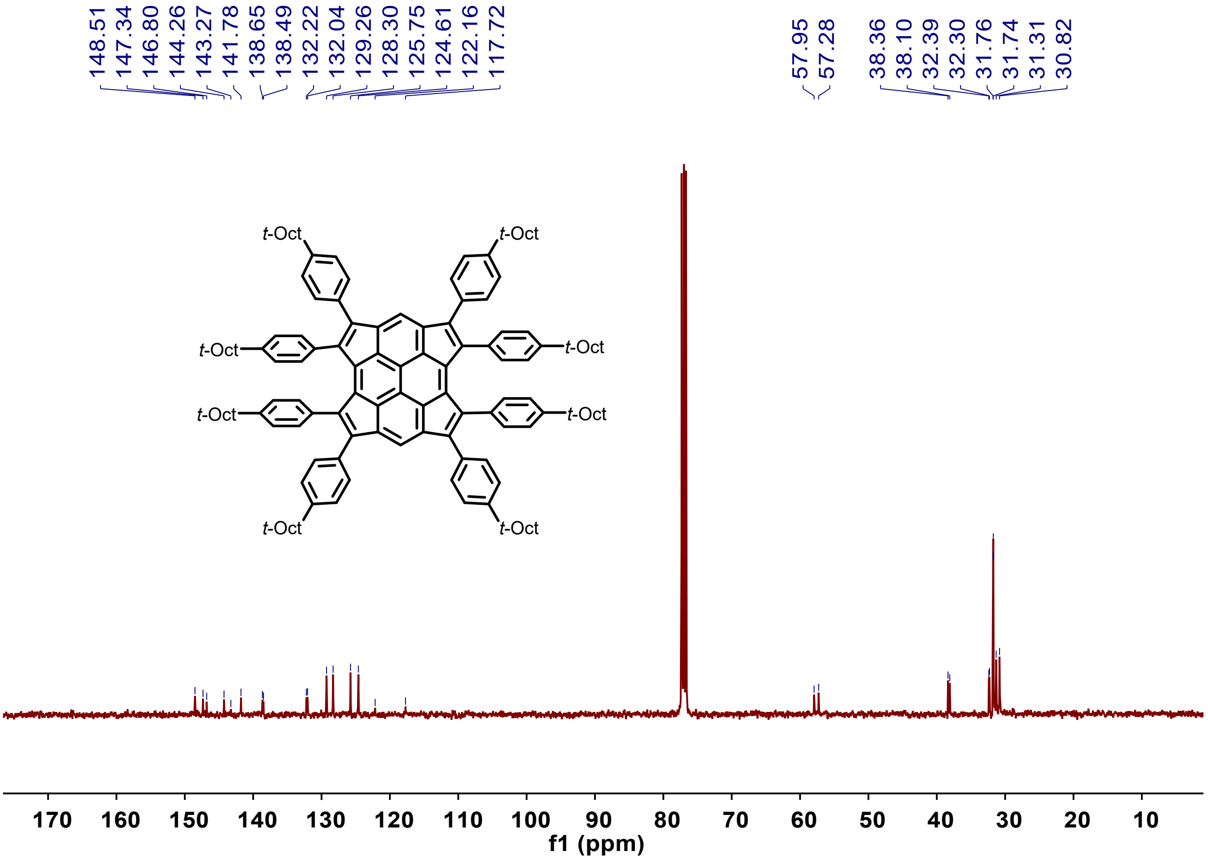

Fig. S27 ¹³C NMR spectrum of **1** (Chloroform-*d*, 101 MHz, 298 K).

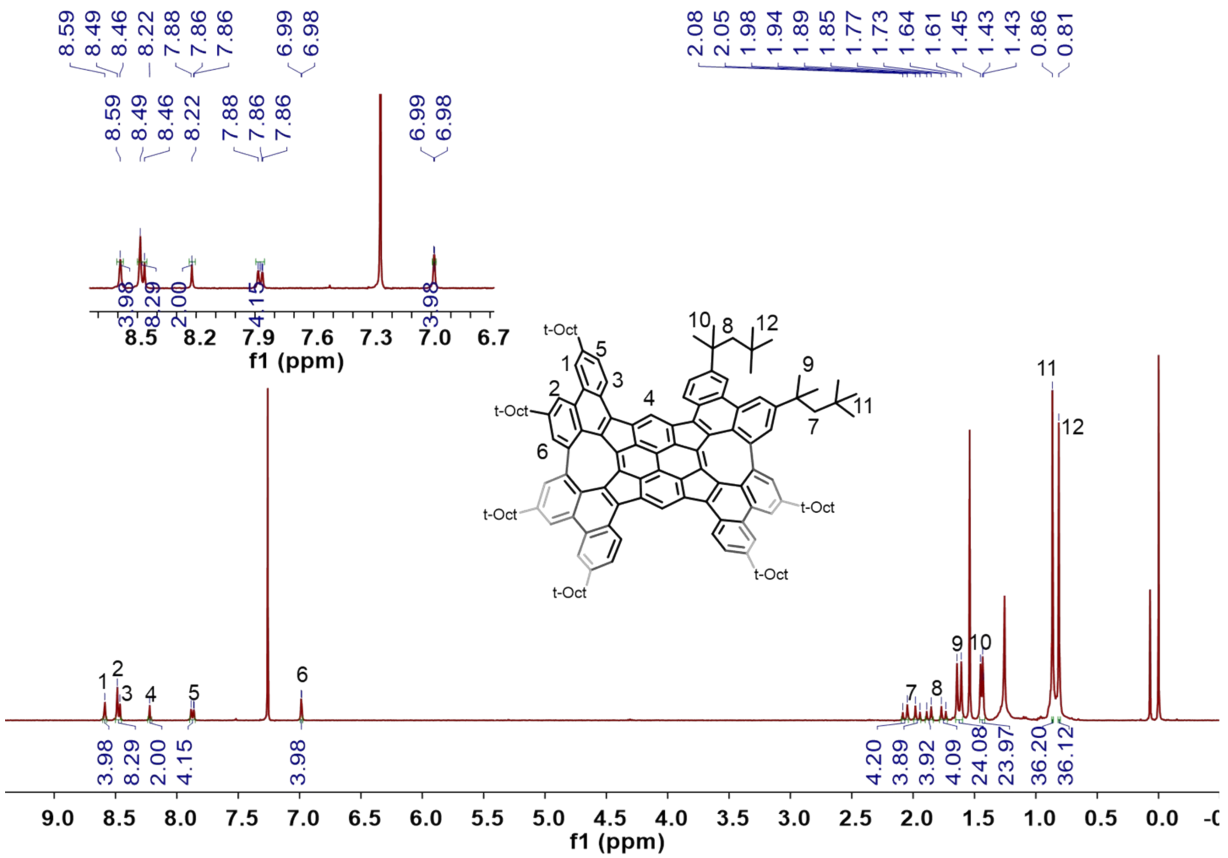

Fig. S28 ^{1}H NMR spectrum of $\mathbf{2_{meso}}$ (Chloroform-*d*, 400 MHz, 298 K).

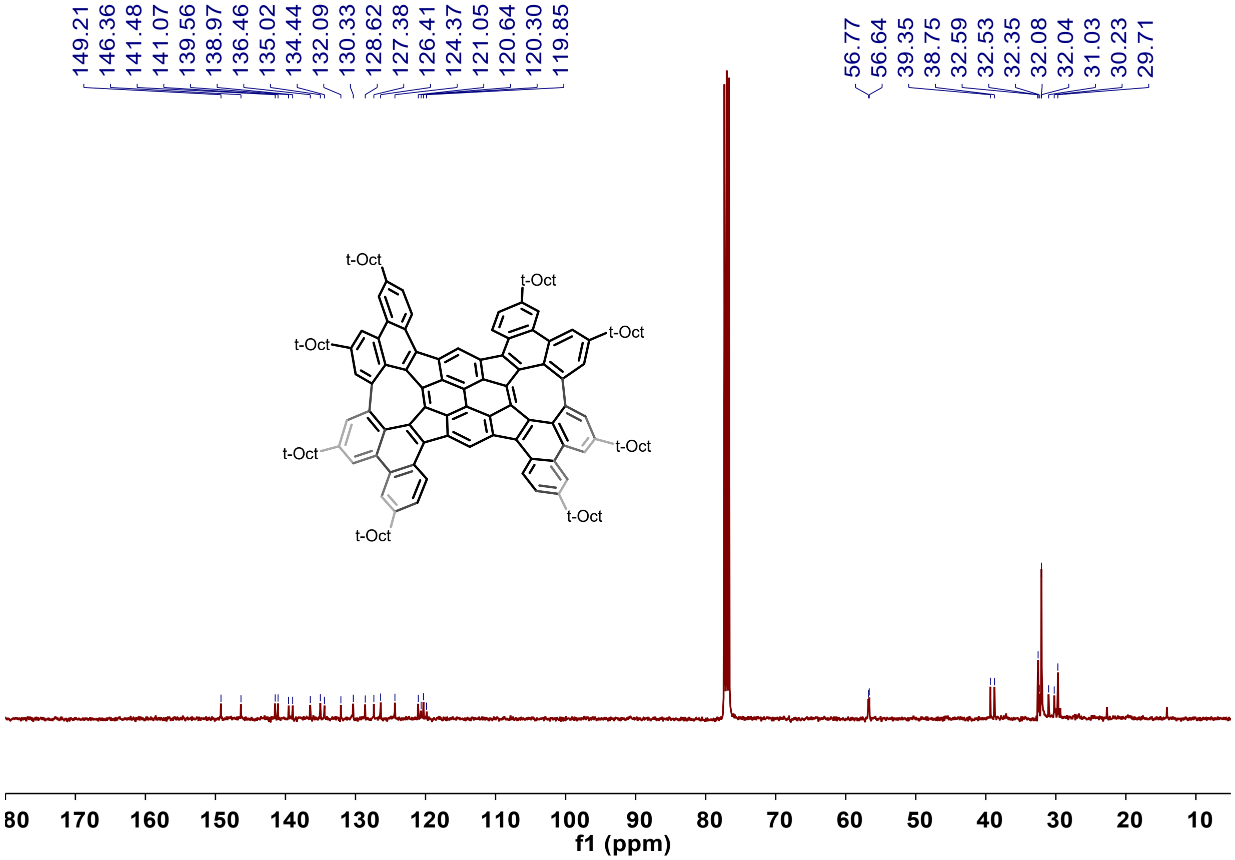

Fig. S29 ^{13}C NMR spectrum of $\mathbf{2_{meso}}$ (Chloroform-*d*, 101 MHz, 298 K).

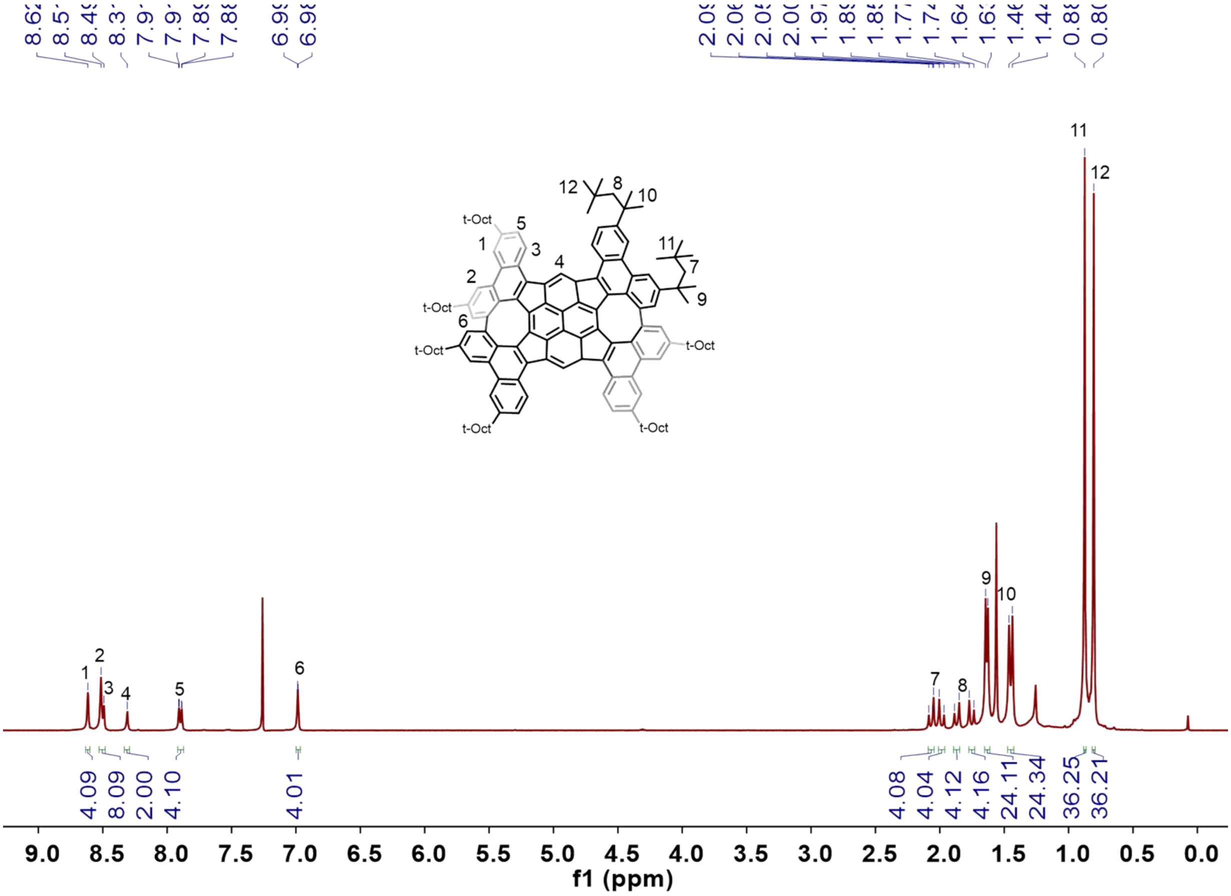

Fig. S30 ^{1}H NMR spectrum of $\mathbf{2_{rac}}$ (Chloroform-*d*, 400 MHz, 298 K).

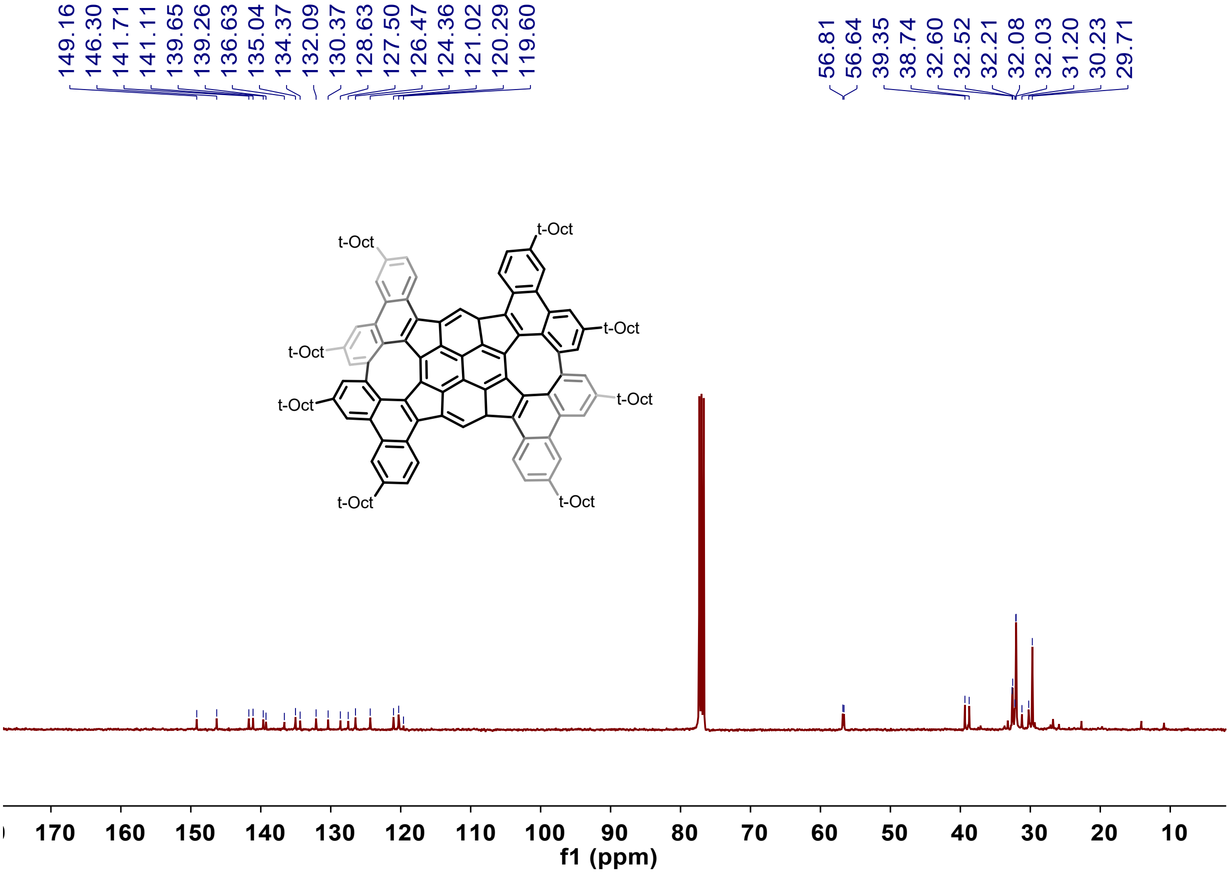

Fig. S31 ^{13}C NMR spectrum of $\mathbf{2_{rac}}$ (Chloroform-*d*, 101 MHz, 298 K).

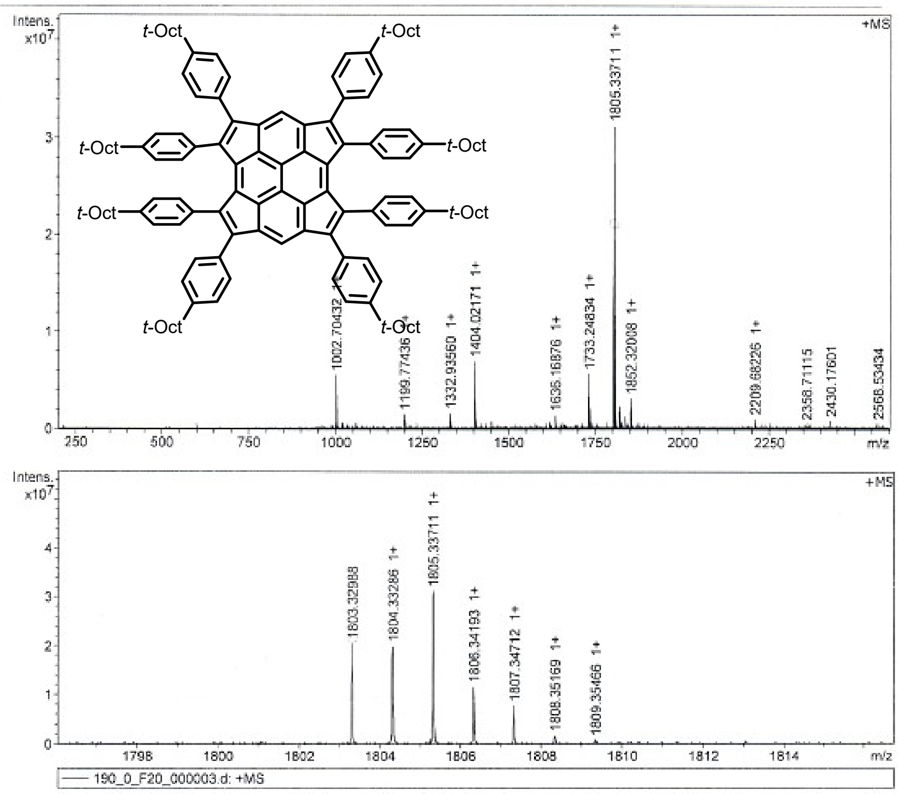

Meas. m/z	#	Ion Formula	Score	m/z	err [ppm]	Mean err [ppm]	mSigma	rdb	e⁻ Conf	N-Rule
1803.329878	1	C136H170	100.00	1803.329707	-0.1	-0.5	173.7	52.0	odd	ok

Fig. S32 HR-MALDI-TOF spectrum of **1**.

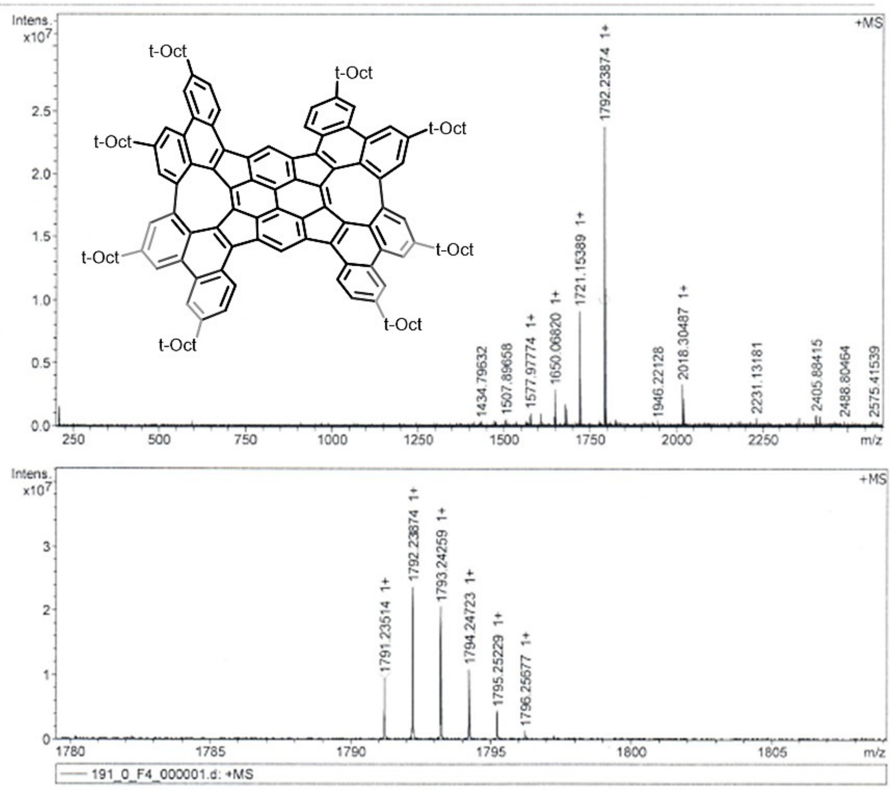

Meas. m/z	#	Ion Formula	Score	m/z	err [ppm]	Mean err [ppm]	mSigma	rdb	e⁻ Conf	N-Rule
1791.235140	1	C136H158	100.00	1791.235806	0.4	-0.1	119.2	58.0	odd	ok

Fig. S33 HR-MALDI-TOF spectrum of $\mathbf{2}_{\mathbf{meso}}$.

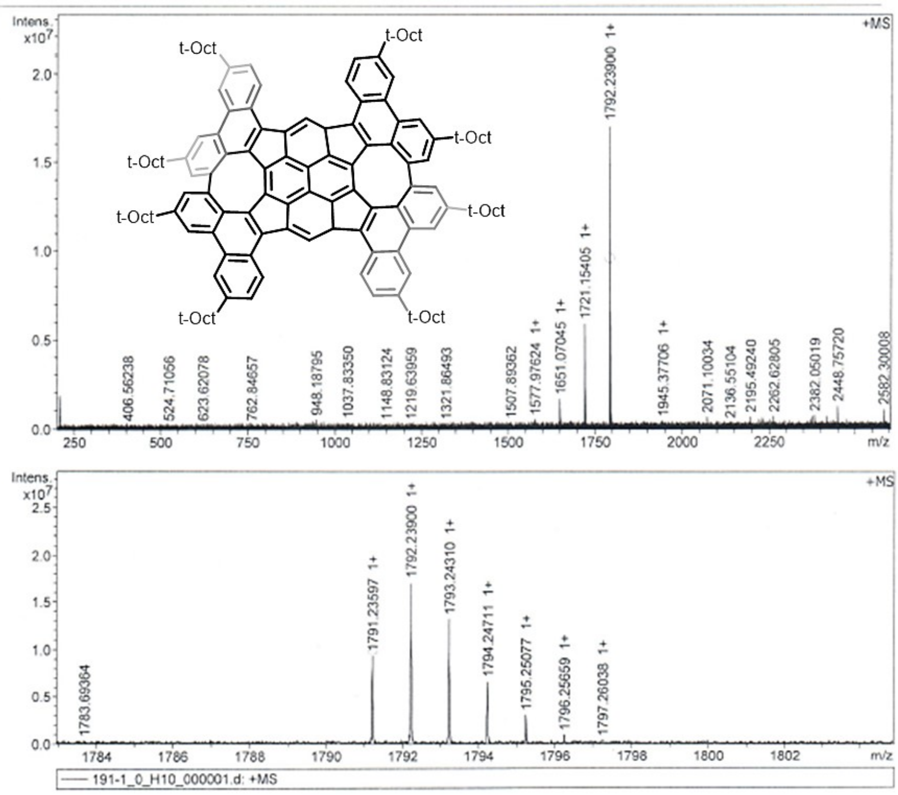

Meas. m/z	#	Ion Formula	Score	m/z	err [ppm]	Mean err [ppm]	mSigma	rdb	e⁻ Conf	N-Rule
1791.235972	1	C136H158	100.00	1791.235806	0.1	-0.2	52.8	58.0	odd	ok

Fig. S34 HR-MALDI-TOF spectrum of $\mathbf{2}_{\mathbf{rac}}$.

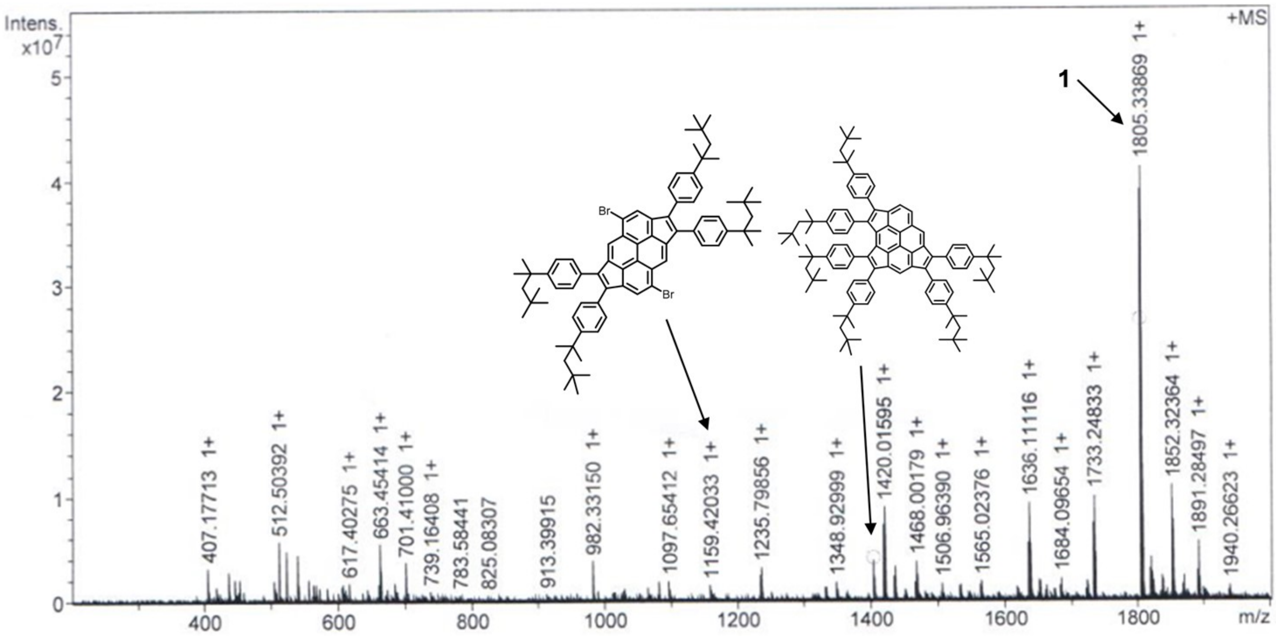

Fig. S35 HR-MALDI-TOF spectrum of two intermediates in the cyclopentannulation reaction.

9. References

[1] C. D. Eversloh, Y. Avlasevich, C. Li and K. Müllen, *Chem. Eur. J.* 2011, **17**, 12756.

[2] T. S. Carter, T. J. Mooibroek, P. F. N. Stewart, M. P. Crump, M. C. Galan and A. P. Davis. *Angew. Chem. Int. Ed.* 2016, **55**, 9311.

[3] M. J. Mio, L. C. Kopel, J. B. Braun, T. L. Gadzikwa, , K. L. Hull, R. G. Brisbois, C. J. Markworth, and P. A. Grieco.

Org. Lett., 2002, **4**, 3199.

[4] Gaussian 09, Revision D.01, M. J. Frisch, G. W. Trucks, H. B. Schlegel, G. E. Scuseria, M. A. Robb, J. R. Cheeseman, G. Scalmani, V. Barone, B. Mennucci, G. A. Petersson, H. Nakatsuji, M. Caricato, X. Li, H. P. Hratchian, A. F. Izmaylov, J. Bloino, G. Zheng, J. L. Sonnenberg, M. Hada, M. Ehara, K. Toyota, R. Fukuda, J. Hasegawa, M. Ishida, T. Nakajima, Y. Honda, O. Kitao, H. Nakai, T. Vreven, J. A. Montgomery, Jr., J. E. Peralta, F. Ogliaro, M. Bearpark, J. J. Heyd, E. Brothers, K. N. Kudin, V. N. Staroverov, T. Keith, R. Kobayashi, J. Normand, K. Raghavachari, A. Rendell, J. C. Burant, S. S. Iyengar, J. Tomasi, M. Cossi, N. Rega, J. M. Millam, M. Klene, J. E. Knox, J. B. Cross, V. Bakken, C. Adamo, J. Jaramillo, R. Gomperts, R. E. Stratmann, O. Yazyev, A. J. Austin, R. Cammi, C. Pomelli, J. W. Ochterski, R. L. Martin, K. Morokuma, V. G. Zakrzewski, G. A. Voth, P. Salvador, J. J. Dannenberg, S. Dapprich, A. D. Daniels, O. Farkas, J. B. Foresman, J. V. Ortiz, J. Cioslowski, and D. J. Fox, Gaussian, Inc., Wallingford CT, 2013.

[5] T. Lu, and F. Chen, *J. Comput. Chem*, 2012, **33**, 580.

[6] T. Lu, and Q. Chen, *J. Comput. Chem*. 2022, **43**, 539.

[7] W. Humphrey, A. Dalke and K. Schulten, *J. Mol. Graph*, 1996, **14**, 33.

[8] (a) Z. Chen, C. S. Wannere, C. Corminboeuf, R. Puchta, and P. v. R. Schleyer, *Chem. Rev*. 2015, **105**, 3842. (b) H. Fallah-Bagher-Shaidaei, S. S. Wannere, C. Corminboeuf, R. Puchta, and P. v. R. Schleyer, *Org. Lett*. 2006, **8**, 863.

[9] D. Geuenich, K. Hess, F. Köhler and R. Herges, *Chem. Rev*. 2005, **105**, 3758.

[10] P. Thordarson, *Chem. Soc. Rev*, 2011, **40**, 1305. http://www.supramolecular.org.
